# Patterns of mortality by occupation in the UK, 1991–2011: a comparative analysis of linked census and mortality records

**DOI:** 10.1016/S2468-2667(17)30193-7

**Published:** 2017-10-23

**Authors:** Srinivasa Vittal Katikireddi, Alastair H Leyland, Martin McKee, Kevin Ralston, David Stuckler

**Affiliations:** a MRC/CSO Social and Public Health Sciences Unit, University of Glasgow, Glasgow, UK; b London School of Hygiene & Tropical Medicine, London, UK; c School of Social and Political Science, Chrystal MacMillan Building, University of Edinburgh, Edinburgh, UK; d Dondena Research Centre and Department of Policy Analysis and Public Management, University of Bocconi, Milan, Italy

## Abstract

**Background:**

Detailed assessments of mortality by occupation are scarce. We aimed to assess mortality by occupation in the UK, differences in rates between England and Wales and Scotland, and changes over time in Scotland.

**Methods:**

We analysed adults of working age (20–59 years) using linked census and death records. Main occupation was coded into more than 60 groups in the 2001 census, with mortality follow-up until Dec 31, 2011. Comparable occupation data were available for Scotland in 1991, allowing assessment of trends over time. We calculated age-standardised all-cause mortality rates (per 100 000 person-years), stratified by sex. We used Monte Carlo simulation to derive p values and 95% CIs for the difference in mortality over time and between England and Wales and Scotland.

**Findings:**

During 4·51 million person-years of follow-up, mortality rates by occupation differed by more than three times between the lowest and highest observed rates in both men and women. Among men in England and Wales, health professionals had the lowest mortality (225 deaths per 100 000 person-years [95% CI 145–304]), with low rates also shown in managers and teachers. The highest mortality rates were in elementary construction (701 deaths per 100 000 person-years [95% CI 593–809]), and housekeeping and factory workers. Among women, teachers and business professionals had low mortality, and factory workers and garment trade workers had high rates. Mortality rates have generally fallen, but have stagnated or even increased among women in some occupations, such as cleaners (337 deaths per 100 000 person years [95% CI 292–382] in 1991, rising to 426 deaths per 100 000 person years in 2001 [371–481]). Findings from simulation models suggested that if mortality rates by occupation in England and Wales applied to Scotland, 631 fewer men (95% CI 285–979; a 9·7% decrease) and 273 fewer women (26–513; 6·7% decrease) of working age would die in Scotland every year. Excess deaths in Scotland were concentrated among lower skilled occupations (eg, female cleaners).

**Interpretation:**

Mortality rates differ greatly by occupation. The excess mortality in Scotland is concentrated among low-skilled workers and, although mortality has improved in men and women in most occupational groups, some groups have experienced increased rates. Future research investigating the specific causes of death at the detailed occupational level will be valuable, particularly with a view to understanding the health implications of precarious employment and the need to improve working conditions in very specific occupational groups.

**Funding:**

None.

## Introduction

Employment has been long established as a fundamental determinant of health.^1^ Mortality rates by occupation were first calculated in the UK in 1851, following the country's second ever census.^2^ Since then, regular decennial reports have provided updated estimates, with the last published in the 1970s.^3^, ^4^, ^5^ Similar analyses from other countries are rare and typically based on broad occupational categories.^6^, ^7^ Ongoing changes in the labour market make a reassessment of mortality by occupation timely. In particular, trends in the job market (such as the rise of so-called zero-hours contracts) could adversely affect health and health inequalities.^8^, ^9^, ^10^ It cannot be assumed that patterns identified in the 1970s, which still underpin our contemporary understanding, continue to apply.

There has been a discernible shift away from mortality by occupation, with more recent research on health inequalities focusing on where people live, on the basis of area-based deprivation measures (such as the Index of Multiple Deprivation). Some research does use social class,^11^, ^12^ but although this measure is based on occupation, it indicates the social position of an individual rather than their specific occupation.^13^ Social class therefore often combines fairly heterogeneous groups into typically fewer than eight categories.^14^, ^15^ Research into mortality by occupation has focused on proportional mortality ratios, which identify the causes of death that are over-represented in specific occupations.^16^, ^17^ Such research is helpful in detecting what diseases are most likely to present in different groups, but does not provide information about how absolute mortality rates differ between occupations and is therefore of little use to inform broad policy choices (as opposed to cause-specific remedies within occupational groups).^18^

**Research in context**

**Evidence before this study**

We searched MEDLINE and Embase on May 19, 2015, to identify studies that reported mortality rates by occupation (from inception and limiting to English-language publications). Search terms included “occupation”, “job”, “mortality”, and “deaths”. Technological and societal developments have led to major changes in the nature of many occupations in recent decades, yet understanding of occupational patterns of mortality in the UK still relies largely on studies done in the 1980s. Internationally, mortality rates by occupation have been reported in South Korea and New Zealand, but these studies are based on relatively few occupational categories. No recent studies that reported detailed mortality rates by occupation were identified. Similarly, mortality by occupation across the UK has not been compared in the past few decades.

**Added value of this study**

Mortality rates continue to show very large variation between occupations among the working-age population of the UK. Among men, health professionals, managers, and teachers had particularly low age-standardised mortality rates. The occupational groups with the highest mortality were elementary construction, housekeeping, and factory workers. Among women, teachers and business professionals had low mortality, with high rates among factory workers and those in the garment trade. Analyses based on Scottish data showed that mortality rates have reduced over time in most occupational groups, but this was not the case for all groups, with some that had high mortality rates in the 1990s experiencing little improvement or even increased mortality rates.

**Implications of all the available evidence**

Mortality rates by occupation differ by more than three times between the lowest and highest observed rates in both men and women in the UK. Although mortality has improved in most occupational groups, it has increased in others; reasons for this finding require investigation. Future research should consider the importance of reporting mortality by detailed occupation, which might provide an opportunity to understand the implications of increasing precariousness of employment and the need to improve working conditions in very specific occupational groups.

Large health differences exist across the countries of the UK.^19^ Mortality in Scotland is consistently higher than elsewhere in the UK, a phenomenon not accounted for by socioeconomic deprivation.^20^ Some research has suggested that this excess mortality occurs across the socioeconomic spectrum, but studies so far have relied on broad classifications of social class, household socioeconomic position, or area-based deprivation.^21^, ^22^, ^23^ Alternative work, also applying aggregated measures, has found that Scotland's excess mortality disproportionately affects the least advantaged.^24^ More detailed measures of occupation, based on classification by skill level, might better explain differences in mortality between Scotland and England, because of the more accurate classification of the population.

We therefore assessed mortality by detailed occupational groups in each part of the UK (England and Wales, Scotland, and Northern Ireland), differences in rates between England and Wales and Scotland, and changes over time in Scotland.

## Methods

### Data sources

We studied representative populations of adults of working age (20–59 years at baseline) in the UK, drawn from random samples of the 2001 census. We used three different data sources for different parts of the UK: the Office for National Statistics (ONS) Longitudinal Study provides a 1% sample of the population of England and Wales,^25^ the Scottish Longitudinal Study provides a 5·3% sample of the Scottish population,^26^ and the Northern Ireland Mortality Study (NIMS) includes 100% of the population of Northern Ireland.^27^ We analysed census data linked to mortality records to create three separate cohorts. For England and Wales and Scotland, deaths from throughout the UK were available from the ONS and National Records Scotland. Because the follow-up process for deaths in Northern Ireland differed, with deaths occurring outside of Northern Ireland unavailable, deaths for Northern Ireland are reported in the appendix. Occupation coded in a comparable manner was available from the 1991 census in Scotland only.

For all three datasets, we had no access to identifiable individual-level data, with all data derived from linkages that were anonymised before handover. Ethics approval was not required, but formal applications were reviewed by each data holder.

### Exposure measures

Occupation was self-reported in the 2001 census, in response to the question “What is the full title of your main job?” The Standard Occupational Classification (SOC) 2000 codes are used by statistical agencies in the UK and elsewhere to classify occupations into meaningful groups, on the basis of comparable levels of training, prestige, and economic reward.^28^ Individuals not reporting an occupation were categorised as a separate group for the analysis (which could include students and homemakers, depending on their self-assessment). The SOC coding system is hierarchical, with four digit codes being the most detailed and one digit codes the least. Due to disclosure control restrictions, we are unable to report occupational groups that included fewer than ten deaths during follow-up. Our analysis therefore generally used three digit codes. When this criterion was not met, we combined groups within the hierarchical categories (appendix pp 2–13).

We sought to ensure that comparable occupational groups were used for analysis across all three datasets, and that codes were as detailed as possible. Because of differences in the jobs held by gender, and the need to minimise disclosure risk, we used different SOC groupings for men and women. Employment status by occupational group could not be considered within the analysis for disclosure control reasons and because of differences in the coding of variables. Therefore, we based the exposure measure on the person's self-reported last main job, rather than the job an individual was actually doing on the date of the census.

### Statistical analysis

Because of data security restrictions, analysis was done separately for each dataset on a standalone computer. For all three datasets, occupational groups were categorised on the basis of responses in the 2001 census and followed up for all-cause mortality until Dec 31, 2011. Mortality rates were calculated per 100 000 person-years, stratified by sex, and directly standardised with the WHO European standard population (5 year age bands) of 2013. To assess trends over time, we assessed mortality by occupation for the 1991 census sample in Scotland, followed up until Dec 31, 2001.

Since the role of chance could not be directly tested within a single statistical model across the separate datasets, we used Monte Carlo simulation to derive p values and 95% CIs for the difference in mortality over time and between England and Wales and Scotland. Simulation of 10 000 mortality rates by occupation was done based on the mean and standard error of the age-standardised rate for each occupation in England and Wales and Scotland. Simulated rates were compared across countries for each simulation, and the 2·5% and 97·5% quantiles of the difference in rates calculated.

To aid interpretation of the public health importance of our findings, we simulated the number of expected deaths in England and Wales and Scotland under different scenarios. We first retrieved information about the age–sex structure of the countries. We defined a baseline scenario as the number of deaths currently expected. Since the occupational structure is observed for only samples of the census, and there were no estimated mortality rates by occupation for a small part of the population, we first created a realistic synthetic population for each geography. To do so, we calculated the number of people expected in each occupational group, assuming the person-years observed in each of the random samples were reflected across the working-age population of their respective geographies in 2001. We then applied observed occupation-specific mortality rates for each geography to calculate the number of expected deaths per year under the baseline scenario. We then modelled anticipated deaths under two alternative scenarios: first, by applying the occupation-specific mortality rates for England and Wales to Scotland, and second by applying the occupation-specific rates for Scotland to England and Wales. These two scenarios provide estimates of how many deaths could be averted within Scotland if occupation-specific mortality rates for England and Wales applied, and how many excess deaths would occur if England and Wales experienced Scottish rates. To estimate 95% CIs, we did Monte Carlo modelling with 10 000 samples. Disclosure rules of the ONS Longitudinal Study and Scottish Longitudinal Study meant that we were unable to use age-specific and occupation-specific numbers of deaths in the Monte Carlo simulations. For this reason our simulation analyses were based on normal approximations to the age-standardised mortality rates rather than the more appropriate Poisson distribution. We did simulation modelling with R (version 3.3.1) and analyses in the safe settings holding the individual-level data with Stata version 13.2.

### Data sharing

The ONS Longitudinal Study, the Scottish Longitudinal Study, and NIMS are available to researchers by application from the data holders.

### Role of the funding source

There was no funding source for this study. The corresponding author had full access to all the data in the study and had final responsibility for the decision to submit for publication.

## Results

The ONS Longitudinal Study and Scottish Longitudinal Study provided 4·51 million person-years of follow-up (2·31 million person-years for women and 2·20 million person-years for men). There were large differences in mortality by occupation in all parts of the UK, with differences of more than three times between the lowest and highest observed rates in both men and women in all three datasets. The largest differences were in Scotland. Patterns of high and low rates were broadly similar across the UK (Table 1, Table 2; appendix 14–21), in view of the imprecise estimation of rates within specific groups.

**Table 1:** Mortality by occupation in England and Wales in men

	**Rank**	SOC code*	**Person-years**	**Mortality rate (95% CI)**†
Health professionals	1	221	12 873	225 (145–304)
Business and public service professionals	2	24	41 276	228 (182–274)
Functional managers	3	113	61 089	233 (194–273)
Financial institution and office managers	4	115	21 375	234 (170–299)
Corporate managers and directors	5	11	13 934	250 (175–326)
Teaching professionals	6	231	36 736	262 (210–313)
Production managers	7	112	55 718	265 (219–311)
Protective service occupations	8	331	28 834	265 (190–340)
Information and communication technology professionals	9	213	31 489	267 (187–348)
Business and finance associate professionals	10	353	25 992	269 (198–339)
Science and engineering technicians	11	311	23 177	270 (199–341)
Managers in distribution, storage, and retailing	12	116	44 677	277 (226–327)
Engineering professionals	13	212	35 715	282 (226–338)
Draughts persons and building inspectors	14	312	5009	282 (137–428)
Health associate professionals	15	321	9248	288 (171–404)
Transport associate professionals	16	351	5078	290 (117–464)
Public service and other associate professionals	17	356	13 681	309 (212–405)
Protective service officers	18	117	6365	323 (188–459)
Managers and proprietors in other service industries	19	123	36 544	333 (278–387)
Electrical trades	20	524	41 849	333 (276–389)
Social welfare associate professionals	21	323	5084	334 (166–501)
Leisure and travel service occupations	22	621	6145	345 (165–524)
Administrative occupations: finance	23	412	23 118	347 (265–429)
Secretarial and related occupations	24	421	4784	348 (187–510)
Culture, media, and sports occupations	25	34	32 202	350 (273–428)
Information technology service delivery occupations	26	313	10 582	350 (169–532)
Sales and related associate professionals	27	354	26 967	362 (285–438)
Sales-related occupations	28	712	9552	368 (251–485)
Administrative occupations: government and related organisations	29	411	11 250	373 (268–479)
Customer service occupations	30	721	8397	385 (198–573)
Elementary administration occupations	31	921	22 354	389 (305–473)
Skilled trades not elsewhere classified	32	549	9553	391 (259–524)
Metal machining, fitting, and instrument making trades	33	522	42 963	400 (342–458)
Agricultural trades	34	511	23 913	402 (325–479)
Printing trades	35	542	7140	403 (244–562)
Health care and related personal services	36	611	11 400	413 (287–539)
Administrative occupations: records	37	413	20 129	418 (318–517)
Construction trades	38	531	71 558	419 (370–467)
Plant and machine operatives	39	812	38 091	423 (360–487)
Sales assistants and retail cashiers	40	711	33 239	429 (331–526)
Food preparation trades	41	543	23 061	439 (335–542)
Transport drivers and operatives	42	821	82 677	445 (401–489)
Building trades	43	532	23 344	446 (360–532)
Managers and proprietors in agriculture and services	44	12	23 348	450 (364–536)
Vehicle trades	45	523	23 097	456 (358–553)
Administrative occupations: general	46	415	16 102	461 (342–581)
Hairdressers and related occupations	47	622	2755	492 (226–759)
Assemblers and routine operatives	48	813	25 176	501 (410–593)
Construction operatives	49	814	16 247	505 (399–611)
Process operatives	50	811	30 195	529 (445–614)
Elementary goods storage occupations	51	914	37 887	539 (459–619)
Elementary security occupations	52	924	15 272	544 (433–655)
Mobile machine drivers and operatives	53	822	14 661	556 (435–676)
Elementary sales occupations	54	925	3571	556 (209–903)
Metal forming, welding, and related trades	55	521	16 479	563 (451–675)
Housekeeping occupations	56	623	5202	567 (402–723)
Textiles and garments trades	57	541	3809	569 (330–808)
Elementary cleaning occupations	58	923	20 096	592 (487–696)
Administrative occupations: communications	59	414	2204	604 (246–962)
Elementary agricultural occupations	60	911	8101	623 (441–805)
Elementary personal services occupations	61	922	21 359	650 (510–790)
Elementary process plant occupations	62	913	32 513	672 (576–767)
Elementary construction occupations	63	912	22 988	701 (593–809)
No occupation reported	..	..	39 614	1189 (1014–1364)
All those reporting an occupation‡	..	..	1 457 772	384 (374–394)

SOC=Standard Occupational Classification.

* SOC codes have been modified for disclosure control purposes (appendix pp 2–13).

† Age-standardised mortality rates per 100 000 person-years.

‡ Includes not only SOC categories listed, but also small occupational groups that have been suppressed for disclosure control (equating to a total of 1·2% of all person-years). Therefore, person-years for the SOC codes listed do not total to the last two rows. Source: Office of National Statistics Longitudinal Study.^25^

**Table 2:** Mortality by occupation in England and Wales in women

	**Rank**	SOC code*	**Person-years**	**Mortality rate (95% CI)**†
Culture, media, and sports occupations	1	34	26 903	133 (78–188)
Business and public service professionals	2	24	25 728	159 (104–214)
Teaching professionals	3	231	79 871	180 (152–208)
Science, research, engineering, and technology professionals	4	21	13 446	180 (82–279)
Business and public service associate professionals	5	35	64 710	188 (146–230)
Science and technology associate professionals	6	31	15 796	203 (127–279)
Child care and related personal services	7	612	55 829	204 (157–250)
Corporate managers and directors	8	11	106 025	209 (178–239)
Hairdressers and related occupations	9	622	21 910	209 (142–277)
Health professionals	10	221	10 099	213 (107–318)
Food preparation trades	11	543	18 319	217 (153–281)
Secretarial and related occupations	12	421	112 533	218 (192–244)
Elementary sales occupations	13	925	6775	218 (93–344)
Elementary goods storage occupations	14	914	7984	220 (104–336)
Leisure and travel service occupations	15	621	12 344	222 (116–328)
Managers and proprietors in other service industries	16	123	23 180	227 (162–291)
Customer service occupations	17	721	25 489	230 (153–307)
Health associate professionals	18	321	58 260	230 (191–270)
Administrative occupations: general	19	415	76 601	233 (198–268)
Social welfare associate professionals	20	323	13 795	236 (153–318)
Agricultural trades	21	511	4776	251 (114–389)
Printing trades	22	542	3557	256 (104–409)
Administrative occupations: government and related organisations	23	411	21 080	263 (192–334)
Administrative occupations: finance	24	412	72 532	264 (223–305)
Sales-related occupations	25	712	9852	265 (156–375)
Transport drivers and operatives	26	821	6560	279 (132–426)
Health care and related personal services	27	611	88 120	290 (255–325)
Elementary security occupations	28	924	16 424	301 (222–380)
Sales assistants and retail cashiers	29	711	137 985	305 (275–336)
Housekeeping occupations	30	623	8417	306 (204–407)
Elementary personal services occupations	31	922	59 492	314 (265–363)
Administrative occupations: records	32	413	37 807	333 (271–394)
Process operatives	33	811	17 107	346 (259–433)
Elementary cleaning occupations	34	923	65 159	352 (311–394)
Skilled metal and electrical trades	35	52	4745	367 (184–550)
Administrative occupations: communications	36	414	6727	368 (233–502)
Skilled trades not elsewhere classified	37	549	4170	380 (175–585)
Elementary administration occupations	38	921	12 940	383 (275–492)
Assemblers and routine operatives	39	813	34 561	386 (324–448)
Managers and proprietors in agriculture and services	40	12	23 892	397 (318–476)
Elementary process plant occupations	41	913	30 212	405 (334–476)
Textiles and garments trades	42	541	3810	483 (266–700)
Plant and machine operatives	43	812	8707	517 (370–663)
No occupation reported	..	..	83 370	587 (523–651)
All those reporting an occupation‡	..	..	1 485 036	263 (255–272)

SOC=Standard Occupational Classification.

* SOC codes have been modified for disclosure control purposes (appendix pp 2–13).

† Age-standardised mortality rates per 100 000 person-years.

‡ Includes not only SOC categories listed, but also small occupational groups that have been suppressed for disclosure control (equating to a total of 1·2% of all person-years). Therefore, person-years for the SOC codes listed do not total to the last two rows. Source: Office of National Statistics Longitudinal Study.^25^

For men in England and Wales (table 1), health professionals (comprising medical doctors, dentists, psychologists, pharmacists, opticians, and vets) had the lowest mortality rates in all three datasets. Business and public services professionals (including lawyers, architects, and accountants) also had very low mortality, followed by different groups of managerial staff. Of men in intermediate skilled jobs, those working in electrical trades (including electricians, telecommunications engineers, and computer engineers) had relatively low mortality compared with those in other jobs of a similar skill level. The highest mortality rates were in unskilled construction workers and those working in factories or similar settings (ie, elementary process plant jobs, including packers, canners, fillers, and labourers in foundries). Elementary personal services occupations, which include hospital or hotel porters, kitchen and catering assistants, and bar staff, also had high mortality rates. Occupational groups that included large numbers of people and tended to have high mortality rates were goods storage occupations (which include dockers and other goods handling occupations), process operatives (people involved in the processing of goods, such as food, drink, rubber, and plastics), and transport drivers and operatives (eg, drivers of heavy goods vehicles, taxis, and buses). Administrative and sales jobs had intermediate mortality rates. The highest mortality rates overall were in men who reported no occupation (table 1).

Among women, the lower mortality rates resulted in some variability in the ranking of some smaller occupational groups across the three datasets. The groups with low mortality in England, as well as Northern Ireland and Scotland, included business and public services professionals, teachers and corporate managers, and directors (table 2; appendix pp 16, 17, 20). Nurses and allied health professionals had relatively low mortality, whereas w omen in administrative jobs tended to have intermediate mortality. Of the intermediate skilled jobs, child care and related personal services had relatively low mortality rates compared with jobs of a similar skill level. The highest mortality rates occurred in factory workers, including those working in the textile and garment trade. As with men, women in elementary process plant occupations had high mortality rates. Of the larger occupational groups, assemblers and routine operatives (eg, assemblers of electrical products, inspectors, and routine testers), elementary cleaning occupations, and elementary personal services occupations had high mortality rates. Women reporting an occupation had lower mortality rates than those who did not (table 2).

Rankings of mortality by occupation were generally similar between men and women (appendix pp 21, 22). Some differences were the relatively low mortality risks in women working as hairdressers and in elementary sales compared with men working in these occupations.

Mortality rates in Northern Ireland could not be directly compared with those in other parts of the UK because of under-ascertainment of mortality in the study data; however, the ranking of occupations was generally similar to other geographies (appendix pp 18–20). It was possible to report some specific mortality rates by occupation from Northern Ireland that could not be ascertained in other geographies for disclosure control reasons. For example, research professionals had relatively low mortality rates in both men and women in Northern Ireland (appendix pp 18–20).

Occupational mortality rates tended to be slightly higher in Scotland than in England and Wales (figure). Among both men and women, rates did not seem to differ systematically between Scotland and England and Wales for more highly skilled jobs. By contrast, lower skilled occupations with high mortality rates tended to have even higher rates in Scotland. However, in view of the relatively small sample size for many categories, mortality differed significantly between only a few specific occupational groups.

**Figure:**
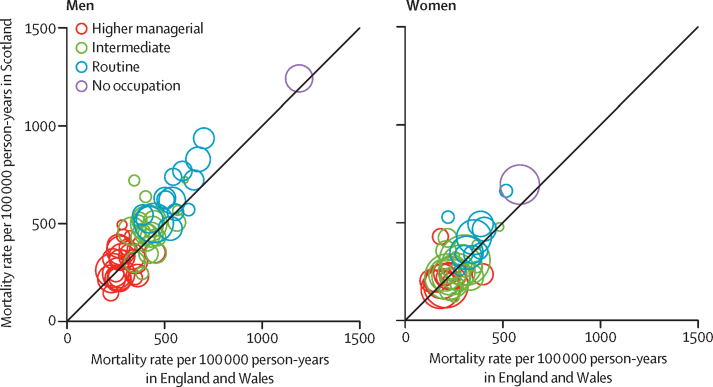
Bubble graphs comparing mortality by occupation between England and Wales and Scotland, 2001–11 Each bubble is proportional to the size of the employment group in England and Wales. Higher managerial occupations were classified as major Standard Occupational Classification [SOC] groups 1–3, intermediate occupations as major SOC groups 4–7, and routine occupations as major SOC groups 8 and 9.

Assessment of trends within Scotland showed that men and women in most occupational groups had reduced mortality; however, mortality rates increased among some occupations (Table 3, Table 4). Mortality in three occupational groups (assemblers and routine operatives, elementary cleaning occupations, and people reporting no job) was high in 1991, and rose over time in women—a pattern that did not seem to be due to chance (Table 3, Table 4).

**Table 3:** Differences in mortality by occupation in Scotland between 1991–2001 and 2001–11 in men

	SOC code*	**Mortality rate (95% CI)**†		**Difference in mortality rates**‡	
		1991 follow-up period	2001 follow-up period	Difference (95% CI)	p value
Corporate managers and directors	11	512 (274 to 749)	244 (117 to 372)	−268 (−538 to −1)	0·05
Production managers	112	246 (176 to 315)	243 (182 to 305)	−3·1 (−95 to 90)	0·94
Functional managers	113	458 (335 to 581)	261 (172 to 351)	−196 (−345 to −43)	0·01
Financial institution and office managers	115	335 (192 to 478)	321 (191 to 451)	−14 (−209 to 181)	0·88
Managers in distribution, storage, and retailing	116	344 (235 to 453)	363 (252 to 474)	19 (−135 to 174)	0·81
Protective service officers	117	393 (153 to 634)	436 (165 to 707)	41 (−328 to 398)	0·82
Managers and proprietors in agriculture and services	12	399 (263 to 535)	353 (242 to 464)	−45 (−218 to 132)	0·61
Managers and proprietors in other service industries	123	397 (299 to 496)	333 (241 to 426)	−64 (−200 to 73)	0·36
Natural and social science professionals	211	340 (61 to 618)	..	..	..
Engineering professionals	212	341 (229 to 452)	333 (234 to 432)	−7·9 (−154 to 142)	0·91
Information and communication technology professionals	213	552 (62 to 1042)	214 (81 to 347)	−341 (−843 to 165)	0·19
Health professionals	221	301 (141 to 461)	145 (50 to 240)	−156 (−342 to 30)	0·10
Teaching professionals	231	299 (220 to 377)	219 (158 to 281)	−79 (−178 to 21)	0·12
Research professionals	232	..	328 (26 to 630)	..	..
Business and public services professionals	24	309 (241 to 404)	218 (149 to 287)	−91 (−207 to 23)	0·12
Science and engineering technicians	311	456 (305 to 608)	314 (206 to 422)	−141 (−325 to 44)	0·13
Draughts persons and building inspectors	312	285 (95 to 474)	494 (212 to 777)	206 (−135 to 542)	0·23
Information technology service delivery occupations	313	..	224 (0 to 456)	..	..
Health associate professionals	321	755 (389 to 1120)	438 (220 to 657)	−316 (−744 to 117)	0·14
Social welfare associate professionals	323	552 (193 to 910)	372 (141 to 603)	−179 (−615 to 245)	0·42
Protective service occupations	331	552 (372 to 732)	386 (255 to 517)	−165 (−384 to 53)	0·14
Culture, media, and sports occupations	34	469 (289 to 650)	307 (187 to 427)	−163 (−381 to 53)	0·14
Transport associate professionals	351	545 (292 to 798)	339 (136 to 543)	−204 (−526 to 123)	0·23
Business and finance associate professionals	353	340 (184 to 496)	229 (125 to 333)	−111 (−298 to 82)	0·24
Sales and related associate professionals	354	419 (285 to 552)	235 (140 to 329)	−186 (−346 to −21)	0·03
Public service and other associate professionals	356	443 (293 to 592)	379 (242 to 517)	−62 (−260 to 142)	0·54
Administrative occupations: government and related organisations	411	513 (330 to 695)	521 (343 to 699)	10 (−248 to 266)	0·94
Administrative occupations: finance	412	365 (208 to 521)	303 (161 to 446)	−62 (−275 to 150)	0·58
Administrative occupations: records	413	556 (390 to 723)	346 (220 to 472)	−209 (−422 to 4)	0·05
Administrative occupations: communications	414	846 (168 to 1524)	724 (39 to 1410)	−119 (−1090 to 841)	0·81
Administrative occupations: general	415	338 (142 to 534)	430 (242 to 618)	91 (−181 to 364)	0·50
Secretarial and related occupations	421	..	..	..	..
Agricultural trades	511	447 (352 to 541)	447 (350 to 544)	−0·4 (−135 to 136)	1·00
Metal forming, welding, and related trades	521	657 (508 to 807)	507 (370 to 643)	−151 (−358 to 53)	0·14
Metal machining, fitting, and instrument making trades	522	556 (457 to 656)	431 (344 to 518)	−126 (−259 to 5)	0·06
Vehicle trades	523	474 (304 to 644)	351 (231 to 471)	−124 (−329 to 82)	0·24
Electrical trades	524	404 (316 to 492)	462 (371 to 552)	59 (−66 to 186)	0·37
Construction trades	531	536 (457 to 614)	501 (425 to 577)	−34 (−143 to 75)	0·55
Building trades	532	572 (432 to 711)	513 (380 to 646)	−58 (−248 to 136)	0·55
Textiles and garments trades	541	..	..	..	..
Printing trades	542	314 (108 to 520)	638 (315 to 962)	325 (−67 to 704)	0·10
Food preparation trades	543	641 (439 to 843)	485 (351 to 619)	−157 (−393 to 87)	0·20
Skilled trades not elsewhere classified	549	289 (76 to 503)	461 (219 to 704)	172 (−145 to 493)	0·29
Health care and related personal services	611	452 (213 to 691)	434 (266 to 603)	−18 (−314 to 276)	0·91
Animal care services	613	..	1125 (127 to 2123)	..	..
Leisure and travel service occupations	621	515 (195 to 835)	721 (377 to 1065)	204 (−268 to 675)	0·39
Housekeeping occupations	623	623 (380 to 866)	568 (318 to 818)	−56 (−400 to 291)	0·76
Sales assistants and retail cashiers	711	331 (159 to 503)	444 (283 to 605)	112 (−123 to 343)	0·35
Sales-related occupations	712	573 (380 to 767)	509 (303 to 715)	−64 (−353 to 216)	0·66
Customer service occupations	721	..	247 (49 to 444)	..	..
Process operatives	811	576 (456 to 696)	476 (359 to 592)	−99 (−266 to 67)	0·24
Plant and machine operatives	812	560 (451 to 670)	521 (410 to 631)	−41 (−198 to 114)	0·61
Assemblers and routine operatives	813	611 (439 to 784)	630 (495 to 785)	19 (−209 to 253)	0·88
Construction operatives	814	533 (399 to 667)	623 (472 to 774)	89 (−116 to 292)	0·39
Transport drivers and operatives	821	644 (570 to 718)	503 (440 to 566)	−141 (−239 to −44)	0·01
Mobile machine drivers and operatives	822	861 (677 to 1045)	559 (410 to 708)	−303 (−541 to −64)	0·01
Elementary agricultural occupations	911	574 (404 to 744)	572 (390 to 754)	−1·4 (−251 to 246)	1·00
Elementary construction occupations	912	815 (656 to 973)	937 (771 to 1103)	122 (−106 to 351)	0·29
Elementary process plant occupations	913	614 (463 to 766)	829 (666 to 992)	215 (−10 to 439)	0·06
Elementary goods storage occupations	914	612 (481 to 743)	619 (486 to 752)	6·5 (−182 to 191)	0·94
Elementary administration occupations	921	820 (610 to 1030)	545 (390 to 700)	−276 (−535 to −14)	0·04
Elementary personal services occupations	922	723 (488 to 958)	724 (520 to 927)	1·2 (−311 to 312)	0·99
Elementary cleaning occupations	923	778 (587 to 969)	770 (605 to 934)	−7·3 (−259 to 244)	0·96
Elementary security occupations	924	672 (503 to 842)	740 (560 to 919)	69 (−180 to 319)	0·58
No job reported	1000	1494 (1391 to 1597)	1242 (1131 to 1352)	−252 (−402 to −99)	0·002

Rates for some occupational groups at one or both timepoints have been suppressed because of disclosure control rules. SOC=Standard Occupational Classification.

* SOC codes have been modified for disclosure control purposes (appendix pp 2–13).

† Age-standardised mortality rates per 100 000 person-years.

‡ We used Monte Carlo simulation to estimate differences, 95% CIs, and p values for rates in 2001–11 versus 1991–2001.

**Table 4:** Differences in mortality by occupation in Scotland between 1991–2001 and 2001–11 in women

	SOC code*	**Mortality rate (95% CI)**†		**Difference in mortality rates**‡	
		1991 follow-up period	2001 follow-up period	Difference (95% CI)	p value
Corporate managers and directors	11	194 (122 to 267)	178 (131 to 225)	−16 (−102 to 71)	0·73
Managers and proprietors in agriculture and services	12	386 (243 to 529)	236 (147 to 325)	−148 (−316 to 18)	0·08
Managers and proprietors in other service industries	123	304 (178 to 429)	243 (148 to 338)	−61 (−216 to 96)	0·44
Science, research, engineering, and technology professionals	21	..	429 (24 to 833)	..	..
Health professionals	221	259 (16 to 502)	215 (63 to 366)	−45 (−336 to 248)	0·76
Teaching professionals	231	220 (164 to 276)	160 (122 to 197)	−61 (−130 to 7)	0·08
Business and public service professionals	24	221 (80 to 361)	201 (109 to 292)	−21 (−189 to 148)	0·80
Science and technology associate professionals	31	332 (166 to 548)	220 (104 to 341)	−115 (−361 to 131)	0·37
Health associate professionals	321	242 (177 to 307)	241 (186 to 296)	−0·3 (−84 to 83)	0·99
Therapists	322	..	148 (28 to 269)	..	..
Social welfare associate professionals	323	356 (169 to 544)	202 (110 to 295)	−155 (−359 to 54)	0·14
Protective service occupations	331	..	570 (42 to 1097)	..	..
Culture, media, and sports occupations	34	340 (32 to 647)	203 (83 to 323)	−136 (−470 to 203)	0·41
Business and public service associate professionals	35	278 (151 to 406)	200 (131 to 269)	−78 (−222 to 70)	0·30
Administrative occupations: government and related organisations	411	222 (141 to 303)	238 (154 to 321)	16 (−100 to 130)	0·78
Administrative occupations: finance	412	212 (149 to 274)	249 (190 to 308)	38 (−48 to 125)	0·39
Administrative occupations: records	413	210 (128 to 292)	222 (147 to 297)	13 (−99 to 125)	0·82
Administrative occupations: communications	414	314 (125 to 502)	273 (118 to 428)	−38 (−279 to 210)	0·76
Administrative occupations: general	415	246 (175 to 318)	226 (177 to 276)	−19 (−107 to 69)	0·67
Secretarial and related occupations	421	206 (160 to 253)	223 (180 to 266)	17 (−46 to 81)	0·61
Agricultural trades	511	..	118 (15 to 221)	..	..
Skilled metal and electrical trades	52	..	387 (80 to 694)	..	..
Textiles and garments trades	541	406 (142 to 669)	478 (200 to 756)	72 (−303 to 463)	0·71
Printing trades	542	756 (157 to 1356)	330 (38 to 622)	−429 (−1106 to 230)	0·21
Food preparation trades	543	301 (195 to 407)	422 (299 to 546)	121 (−40 to 284)	0·14
Health care and related personal services	611	288 (221 to 354)	284 (237 to 330)	−4·2 (−86 to 78)	0·93
Child care and related personal services	612	199 (96 to 301)	232 (156 to 309)	33 (−94 to 161)	0·62
Leisure and travel service occupations	621	258 (36 to 480)	228 (99 to 357)	−29 (−284 to 225)	0·83
Hairdressers and related occupations	622	..	356 (185 to 526)	..	..
Housekeeping occupations	623	323 (141 to 506)	254 (129 to 380)	−70 (−290 to 148)	0·54
Sales assistants and retail cashiers	711	279 (231 to 327)	308 (264 to 351)	29 (−34 to 94)	0·38
Sales-related occupations	712	372 (137 to 608)	186 (46 to 325)	−187 (−467 to 89)	0·18
Customer service occupations	721	..	158 (83 to 233)	..	..
Process operatives	811	331 (194 to 467)	338 (218 to 457)	7·2 (−173 to 185)	0·94
Plant and machine operatives	812	481 (141 to 822)	663 (320 to 1006)	185 (−302 to 667)	0·47
Assemblers and routine operatives	813	286 (207 to 365)	494 (399 to 588)	206 (82 to 331)	0·001
Transport drivers and operatives	821	436 (112 to 760)	257 (52 to 462)	−177 (−570 to 196)	0·37
Elementary agricultural occupations	911	425 (50 to 801)	..	..	..
Elementary process plant occupations	913	363 (230 to 497)	467 (338 to 565)	102 (−84 to 286)	0·29
Elementary goods storage occupations	914	521 (195 to 848)	528 (189 to 867)	4·5 (−465 to 477)	0·98
Elementary administration occupations	921	314 (61 to 567)	354 (180 to 528)	41 (−265 to 348)	0·78
Elementary personal services occupations	922	307 (239 to 375)	346 (276 to 415)	38 (−58 to 135)	0·44
Elementary cleaning occupations	923	337 (292 to 382)	426 (371 to 481)	88 (18 to 159)	0·01
Elementary security occupations	924	146 (26 to 265)	339 (178 to 500)	193 (−12 to 397)	0·07
No job reported	1000	597 (557 to 636)	695 (628 to 762)	98 (18 to 174)	0·02

Rates for some occupational groups at one or both timepoints have been suppressed because of disclosure control rules. SOC=Standard Occupational Classification.

* SOC codes have been modified for disclosure control purposes (appendix pp 2–13).

† Age-standardised mortality rates per 100 000 person-years.

‡ We used Monte Carlo simulation to estimate differences, 95% CIs, and p values for rates in 2001–11 versus 1991–2001.

In our simulation analyses, which applied mortality rates from England and Wales to the population of Scotland, we estimated 631 (95% CI 285–979) excess deaths in men (out of 6519 deaths, a 9·7% difference) and 273 (26–513) excess deaths in women (out of 4068 deaths, a 6·7% difference) among the working-age population of Scotland per year (Table 5, Table 6). If Scottish mortality rates by occupation applied across England and Wales, we estimated that 6085 (95% CI 3008 to 9175) more expected deaths would occur in men and 2273 (−165 to 4688) more deaths would occur in women each year (appendix 23–27).

**Table 5:** Expected change in deaths in Scotland among working-age men if mortality rates by occupation for England and Wales applied

	SOC code*	**Difference between rates in England and Scotland (95% CI)**	**p value**	**Expected change in deaths in Scotland (95% CI)**
Corporate managers and directors	11	6·1 (−142 to 155)	0·94	0·6 (−15 to 16)
Production managers	112	21 (−57 to 97)	0·60	8·4 (−23 to 39)
Functional managers	113	−28 (−125 to 71)	0·57	−12 (−53 to 30)
Financial institution and office managers	115	−86 (−235 to 58)	0·25	−12 (−32 to 8)
Managers in distribution, storage, and retailing	116	−87 (−207 to 34)	0·15	−23 (−54 to 9)
Protective service officers	117	−111 (−410 to 183)	0·48	−6·2 (−23 to 10)
Managers and proprietors in agriculture and services	12	97 (−42 to 238)	0·17	21 (−9 to 50)
Managers and proprietors in other service industries	123	−1·2 (−110 to 105)	0·99	−0·4 (−33 to 31)
Engineering professionals	212	−51 (−165 to 62)	0·38	−15 (−49 to 18)
Information and communication technology professionals	213	52 (−104 to 209)	0·51	11 (−21 to 42)
Health professionals	221	79 (−45 to 202)	0·21	10 (−6 to 27)
Teaching professionals	231	43 (−36 to 124)	0·29	14 (−12 to 40)
Business and public service professionals	24	10 (−72 to 92)	0·82	3·7 (−26 to 34)
Science and engineering technicians	311	−43 (−171 to 88)	0·51	−11 (−44 to 22)
Draughts persons and building inspectors	312	−213 (−535 to 105)	0·19	−14 (−35 to 7)
Information technology service delivery occupations	313	127 (−165 to 430)	0·41	12 (−15 to 39)
Health associate professionals	321	−148 (−400 to 99)	0·24	−13 (−36 to 9)
Social welfare associate professionals	323	−37 (−327 to 251)	0·80	−2·4 (−21 to 16)
Protective service occupations	331	−123 (−276 to 30)	0·11	−41 (−93 to 10)
Culture, media, and sports occupations	34	43 (−101 to 186)	0·56	8·9 (−21 to 39)
Transport associate professionals	351	−49 (−324 to 229)	0·71	−3·6 (−24 to 17)
Business and finance associate professionals	353	39 (−86 to 164)	0·54	7·4 (−16 to 31)
Sales and related associate professionals	354	126 (5 to 250)	0·04	27 (1 to 53)
Public service and other associate professionals	356	−70 (−236 to 95)	0·41	−10 (−35 to 14)
Administrative occupations: government and related organisations	411	−148 (−358 to 61)	0·16	−18 (−42 to 7)
Administrative occupations: finance	412	44 (−120 to 210)	0·61	7·2 (−20 to 35)
Administrative occupations: records	413	71 (−90 to 231)	0·39	15 (−19 to 48)
Administrative occupations: communications	414	−123 (−888 to 655)	0·76	−3·0 (−22 to 16)
Administrative occupations: general	415	30 (−189 to 254)	0·80	3·7 (−24 to 32)
Agricultural trades	511	−45 (−167 to 78)	0·48	−17 (−62 to 29)
Metal forming, welding, and related trades	521	55 (−124 to 233)	0·56	11 (−24 to 45)
Metal machining, fitting, and instrument making trades	522	−31 (−135 to 75)	0·56	−13 (−56 to 31)
Vehicle trades	523	106 (−47 to 258)	0·18	22 (−10 to 55)
Electrical trades	524	−128 (−235 to −21)	0·02	−58 (−106 to −10)
Construction trades	531	−83 (−171 to 8)	0·07	−58 (−121 to 6)
Building trades	532	−67 (−224 to 93)	0·41	−15 (−51 to 21)
Printing trades	542	−238 (−605 to 126)	0·19	−13 (−33 to 7)
Food preparation trades	543	−48 (−213 to 121)	0·59	−12 (−54 to 30)
Skilled trades not elsewhere classified	549	−73 (−349 to 201)	0·61	−4·9 (−23 to 14)
Health care and related personal services	611	−20 (−233 to 190)	0·86	−2·4 (−29 to 24)
Leisure and travel service occupations	621	−377 (−761 to 11)	0·06	−25 (−50 to 1)
Housekeeping occupations	623	−1·0 (−302 to 299)	0·996	−0·1 (−21 to 21)
Sales assistants and retail cashiers	711	−16 (−205 to 174)	0·87	−4·4 (−57 to 49)
Sales related occupations	712	−140 (−379 to 97)	0·25	−12 (−33 to 9)
Customer service occupations	721	138 (−138 to 409)	0·33	15 (−15 to 45)
Process operatives	811	53 (−88 to 196)	0·48	14 (−24 to 53)
Plant and machine operatives	812	−98 (−224 to 31)	0·14	−32 (−75 to 10)
Assemblers and routine operatives	813	−128 (−310 to 55)	0·16	−34 (−81 to 14)
Construction operatives	814	−117 (−302 to 66)	0·21	−24 (−61 to 14)
Transport drivers and operatives	821	−58 (−136 to 18)	0·13	−48 (−112 to 14)
Mobile machine drivers and operatives	822	−3·1 (−197 to 184)	0·98	−0·6 (−38 to 36)
Elementary agricultural occupations	911	50 (−208 to 309)	0·71	7·0 (−29 to 43)
Elementary construction occupations	912	−235 (−433 to −37)	0·02	−60 (−111 to −10)
Elementary process plant occupations	913	−157 (−345 to 33)	0·11	−40 (−89 to 9)
Elementary goods storage occupations	914	−80 (−235 to 70)	0·31	−25 (−74 to 22)
Elementary administration occupations	921	−154 (−330 to 21)	0·09	−29 (−62 to 4)
Elementary personal services occupations	922	−73 (−323 to 175)	0·57	−17 (−73 to 40)
Elementary cleaning occupations	923	−179 (−376 to 17)	0·07	−37 (−78 to 4)
Elementary security occupations	924	−196 (−407 to 13)	0·07	−29 (−60 to 2)
No occupation reported	..	−51 (−262 to 154)	0·63	−52 (−269 to 158)
Total	..	..	..	−631 (−979 to −285)

SOC=Standard Occupational Classification.

* SOC codes have been modified for disclosure control purposes (appendix pp 2–13). Source: Office of National Statistics Longitudinal Study.^25^

**Table 6:** Expected change in deaths in Scotland among working-age women if mortality rates by occupation for England and Wales applied

	SOC code*	**Difference between rates in England and Scotland (95% CI)**	**p value**	**Expected change in deaths in Scotland (95% CI)**
Corporate managers and directors	11	30 (−26 to 87)	0·29	23 (−20 to 67)
Managers and proprietors in agriculture and services	12	160 (41 to 276)	0·01	35 (9 to 60)
Managers and proprietors in other service industries	123	−16 (−132 to 98)	0·77	−2·9 (−24 to 18)
Science, research, engineering, and technology professionals	21	−252 (−670 to 159)	0·23	−30 (−79 to 19)
Health professionals	221	−1·3 (−191 to 185)	0·99	−0·1 (−23 to 22)
Teaching professionals	231	20 (−27 to 67)	0·39	15 (−20 to 50)
Business and public service professionals	24	−42 (−150 to 64)	0·45	−10 (−36 to 15)
Science and technology associate professionals	31	−17 (−155 to 124)	0·80	−2·5 (−22 to 18)
Health associate professionals	321	−11 (−80 to 57)	0·76	−6·9 (−53 to 38)
Social welfare associate professionals	323	34 (−92 to 160)	0·60	6·6 (−18 to 32)
Culture, media, and sports occupations	34	−69 (−200 to 62)	0·31	−11 (−33 to 10)
Business and public service associate professionals	35	−11 (−93 to 67)	0·79	−6·2 (−51 to 36)
Administrative occupations: government and related organisations	411	25 (−85 to 132)	0·66	6·5 (−22 to 34)
Administrative occupations: finance	412	15 (−56 to 87)	0·69	8·7 (−33 to 51)
Administrative occupations: records	413	111 (13 to 207)	0·03	38 (4 to 71)
Administrative occupations: communications	414	92 (−115 to 294)	0·38	7·8 (−10 to 25)
Administrative occupations: general	415	6·0 (−56 to 69)	0·86	4·5 (−42 to 52)
Secretarial and related occupations	421	−5·8 (−57 to 45)	0·82	−4·7 (−46 to 37)
Agricultural trades	511	134 (−39 to 302)	0·13	5·5 (−2 to 13)
Skilled metal and electrical trades	52	−19 (−379 to 336)	0·91	−0·6 (−13 to 12)
Textiles and garments trades	541	8·1 (−340 to 361)	0·97	0·3 (−13 to 14)
Printing trades	542	−73 (−397 to 251)	0·67	−2·0 (−11 to 7)
Food preparation trades	543	−205 (−344 to −67)	0·004	−44 (−73 to −14)
Health care and related personal services	611	6·6 (−51 to 66)	0·82	6·0 (−47 to 60)
Childcare and related personal services	612	−29 (−120 to 60)	0·54	−12 (−51 to 26)
Leisure and travel service occupations	621	−5·5 (−173 to 167)	0·95	−0·7 (−23 to 22)
Hairdressers and related occupations	622	−146 (−330 to 37)	0·12	−28 (−62 to 7)
Housekeeping occupations	623	50 (−112 to 211)	0·54	4·9 (−11 to 21)
Sales assistants and retail cashiers	711	−2·3 (−55 to 51)	0·93	−3·2 (−76 to 70)
Sales-related occupations	712	80 (−99 to 256)	0·37	6·2 (−8 to 20)
Customer service occupations	721	71 (−37 to 180)	0·19	20 (−10 to 50)
Process operatives	811	8·8 (−134 to 155)	0·91	1·6 (−24 to 27)
Plant and machine operatives	812	−145 (−517 to 223)	0·44	−6·0 (−21 to 9)
Assemblers and routine operatives	813	−108 (−221 to 2)	0·06	−46 (−94 to 1)
Transport drivers and operatives	821	22 (−232 to 271)	0·86	1·1 (−12 to 14)
Elementary process plant occupations	913	−62 (−206 to 85)	0·41	−13 (−44 to 18)
Elementary goods storage occupations	914	−311 (−668 to 54)	0·09	−14 (−29 to 2)
Elementary administration occupations	921	29 (−180 to 236)	0·78	2·9 (−18 to 23)
Elementary personal services occupations	922	−31 (−115 to 52)	0·48	−22 (−80 to 36)
Elementary cleaning occupations	923	−74 (−144 to −3)	0·04	−64 (−123 to −3)
Elementary security occupations	924	−39 (−218 to 137)	0·68	−2·8 (−16 to 10)
No occupation reported	..	−108 (−200 to −16)	0·02	−135 (−250 to −20)
Total	..	..	..	−273 (−513 to −26)

SOC=Standard Occupational Classification.

* SOC codes have been modified for disclosure control purposes (appendix pp 2–13). Source: Office of National Statistics Longitudinal Study.^25^

## Discussion

Mortality rates by occupation differed by more than three times between the lowest and highest observed rates in men and women in the UK. In men, health professionals, managers, and teachers had particularly low mortality rates, whereas those working in elementary agricultural, construction, and housekeeping jobs had high rates. In women, teachers and business professionals had low mortality, with high rates reported in factory workers and those working in the garment trade. Individuals reporting no occupation had far higher mortality rates than people in all other occupational groups in both men and women. When comparing Scotland to England and Wales, mortality rates were often even higher in health-disadvantaged groups in Scotland, but no consistent differences were shown in people in the most health-advantaged occupations. Findings from simulation models estimate an excess of 631 deaths in men and 273 deaths in women every year in Scotland's working-age population. We studied trends over a 20 year period in Scotland and found that mortality rates have fallen in most occupations, but have remained stagnant or even increased amongst women in some occupational groups.

Our study has several strengths. We analysed three nationally representative administrative datasets. Furthermore, we were able to compare trends in mortality rates by occupation over time because of the availability of similarly categorised occupational information within Scotland. Use of linked data avoids the potential for numerator–denominator bias, a known problem because reports of occupation from death certificates and self-report can differ. Our large sample allowed us to investigate occupational categories in greater detail than is usually possible.

There are several limitations. First, our exposure variable was based on self-reported main occupation at a single timepoint, with follow-up that potentially lasted up to age 70 years. Our findings therefore reflect a respondent's perceived main occupation, but might not reflect their occupation at that or other specific times. We were unable to investigate the role of employment status, which exerts an important influence on mortality,^29^ because of the challenges in carrying out parallel analyses within three different safe settings, which made pooling of data impossible. Although our study is very large, the low risk of mortality in people of working age precludes investigation of cause-specific mortality outcomes within the available administrative data samples. The different structures of occupational categories between men and women prevented formal comparison of results between men and women. Because of the lack of comparability of outcome ascertainment within Northern Ireland, we were unable to compare the rates to those in other parts of the UK. Furthermore, we were unable to study Wales-specific mortality rates. Finally, our analysis is primarily descriptive and cannot assess causal relationships. The quantification of excess mortality in Scotland's working-age population does not imply that improvements in occupational conditions will narrow the gap between countries. Instead, by use of an alternative and more detailed measure of social position, we provide additional insights into the distribution of mortality. Similarly, trends in mortality by occupation reflect both changes in risks and the composition of the occupational groups.

Research into mortality by occupation in other countries has generally been in broad groups. Holmes and colleagues^6^ reported mortality rates by occupation in men in New Zealand according to the nine broadest SOC categories, finding a difference of roughly two times between the highest and lowest rates. Similarly, Lee and colleagues^7^ reported mortality by occupation for the nine most aggregated SOC codes in South Korea and again reported an approximate doubling of mortality between the lowest and highest groups.^7^ Our study provides a much more detailed picture than presented in recent decades, thereby demonstrating larger variations than previously observable.

Mortality by occupation can be considered as being driven by two inter-related factors: the socioeconomic composition of occupational groups, with occupation considered a specific measure of socioeconomic position, and differing exposure to work-related risks and benefits. The labour market has changed radically since publication of the last assessment of mortality by occupation in the UK more than three decades ago, with workers today experiencing very different health outcomes. Monitoring of changes in mortality by occupation is necessary to inform policy responses to address new health risks that arise in response to ongoing and upcoming changes in the labour market. Potential adverse effects of increased job insecurity (reflected by zero-hours contracts) are likely to affect both socioeconomic circumstances (through, for example, low pay and income insecurity) and job stresses (such as a mismatch between job control and work demands) in low-skilled workers—the very groups already experiencing high mortality rates. Looking to the future, there is likely to be a reduction in availability of unskilled (and some skilled) jobs as a consequence of increasing automation. We recorded very high mortality rates among people reporting no occupation in both men and women, with rates in women having potentially increased over time. Reduced availability of low-skilled jobs might therefore herald substantial public health risks.

Mortality rates by occupation are also valuable in guiding prioritisation of scarce health and other resources. There is considerable interest in workplace-based interventions to improve health, but the choice of which occupational groups to target has not been informed by a systematic assessment of need. We have identified specific occupational groups that might benefit from targeted prevention approaches—eg, interventions targeted at male transport drivers who typically have high levels of sedentary time. Employers could play a specific part in mitigation of risks, but the increasingly transient nature of employer–employee relationships might mean that government action is also necessary. Although our findings identify groups at greatest risk, more detailed research is needed to explain why. Such research is likely to require an iterative approach, drilling into the data to understand the potentially complex interactions involved, such as how context (eg, geography) influences the association between occupation and health, and how occupation correlates with different causes of mortality and, by extension, the risk factors involved.

Our study has particular relevance to policy makers in Scotland. There has been considerable concern that health outcomes in Scotland are poorer than elsewhere in western Europe.^30^ Our findings show that excess mortality in Scotland is disproportionately experienced by people in relatively disadvantaged occupations, even when using a detailed measure of occupational skill level. The pattern also varies by gender and is more prominent for men. Our results echo findings that suggest excess mortality in Scotland is particularly concentrated among people living in the most disadvantaged areas.^31^ Efforts in Scotland should focus on meeting the health needs of individuals at the greatest socioeconomic disadvantage.

Future research that investigates the specific causes of death at the detailed occupational level will be valuable, particularly with a view to identifying potential specific interventions to target occupations with the highest mortality risks. Investigation of the inter-relationship between occupational group and employment status will aid understanding of the implications of increasing precariousness of work.^8^, ^9^ Our study has identified very poor, and potentially even worsening, mortality risks for some specific occupational groups. This finding should be a matter of great concern, stimulating further research to understand what is happening.
