# Supplementary material for: Patterns of mortality by occupation in the UK, 1991–2011: a comparative analysis of linked census and mortality records
Source: Lancet Public Health. 2017 Oct 23;2(11):e501–12. doi: 10.1016/S2468-2667(17)30193-7 (PMC5666203; doi:10.1016/S2468-2667(17)30193-7)
Supplement: Supplementary appendix [file mmc1.pdf]

THE LANCET
Public Health

Supplementary appendix

This appendix formed part of the original submission and has been peer reviewed. We post it as supplied by the authors.

Supplement to: Katikireddi SV, Leyland AH, McKee M, Ralston K, Stuckler D. Patterns of mortality by occupation in the UK, 1991–2011: a comparative analysis of linked census and mortality records. *Lancet Public Health* 2017; published online Oct 23. http://dx.doi.org/10.1016/S2468-2667(17)30193-7.

Appendix Contents

Appendix Table 1: Classification of Occupational (SOC) codes 2

Appendix Table 2a: Mortality rates by occupation in Scotland in men 14

Appendix Table 2b: Mortality rates by occupation in Scotland in women 16

Appendix Table 3a: Mortality rates by occupation in Northern Ireland in men 18

Appendix Table 3b: Mortality rates by occupation in Northern Ireland in women 19

Appendix Table 4: Comparison of mortality rates by occupation between men and women in England/Wales for selected occupational groups 20

Appendix Table 5a: Expected change in deaths in England/Wales among working age men if occupational mortality rates for Scotland applied 23

Appendix Table 5b: Expected change in deaths in England/Wales among working age women if occupational mortality rates for Scotland applied 26

Appendix Table 1: Classification of Occupational (SOC) codes

In order to adhere to disclosure control guidelines and ensure adequate statistical power, the classification of SOC codes was modified. The SOC coding system is hierarchical, with the most refined classification provided by four digit unit groups (371 categories). These are nested in 81 minor groups (defined by 3 digit codes) which lie within 25 sub-major groups (defined by two digit codes). At the top of the hierarchy are nine major groups, defined by a single digit code. In order to gain adequate statistical power, we used three digit SOC codes in general.

The below table indicates which SOC codes were used. For some specific categories, additional SOC codes were grouped together and are indicated by numbers in the table below. For example, SOC code 11 in males included SOC codes 111, 114 and 118, but SOC codes 112, 113, 115, 116 and 117 were coded separately.

Major Group	Sub-Major Group	Minor Group	Unit Group	Group Title	Men	Women
1				**MANAGERS, DIRECTORS AND SENIOR OFFICIALS**		
	11			**CORPORATE MANAGERS AND DIRECTORS**	111, 114, 118	X
		111		**Chief Executives and Senior Officials**		
			1111	Senior officials in national government		
			1112	Directors and chief executives of major organisations		
			1113	Senior officials in local government		
			1114	Senior officials of special interest organisations		
		112		**Production Managers**	X	
			1121	Production, works and maintenance managers		
			1122	Managers in construction		
			1123	Managers in mining and energy		
		113		**Functional Managers**	X	
			1131	Financial managers and chartered secretaries		
			1132	Marketing and sales managers		
			1133	Purchasing managers		
			1134	Advertising and public relations managers		
			1135	Personnel, training and industrial relations managers		
			1136	Information and communication technology managers		
			1137	Research and development managers		
		114		**Quality And Customer Care Managers**		
			1141	Quality assurance managers		
			1142	Customer care managers		
		115		**Financial Institution And Office Managers**	X	
			1151	Financial institution managers		
			1152	Office managers		
		116		**Managers In Distribution, Storage And Retailing**	X	
			1161	Transport and distribution managers		
			1162	Storage and warehouse managers		

			1163	Retail and wholesale managers		
		117		**Protective Service Officers**	X	
			1171	Officers in armed forces		
			1172	Police officers (inspectors and above)		
			1173	Senior officers in fire, ambulance, prison and related services		
			1174	Security managers		
		118		**Health And Social Services Managers**		
			1181	Hospital and health service managers		
			1182	Pharmacy managers		
			1183	Healthcare practice managers		
			1184	Social services managers		
			1185	Residential and day care managers		
	12			**MANAGERS AND PROPRIETORS IN AGRICULTURE AND SERVICES**	121+122	121+122
		121		**Managers In Farming, Horticulture, Forestry And Fishing**		
			1211	Farm managers		
			1212	Natural environment and conservation managers		
			1219	Managers in animal husbandry, forestry and fishing n.e.c.		
		122		**Managers And Proprietors In Hospitality And Leisure Services**		
			1221	Hotel and accommodation managers		
			1222	Conference and exhibition managers		
			1223	Restaurant and catering managers		
			1224	Publicans and managers of licensed premises		
			1225	Leisure and sports managers		
			1226	Travel agency managers		
		123		**Managers And Proprietors In Other Service Industries**	X	X
			1231	Property, housing and land managers		
			1232	Garage managers and proprietors		
			1233	Hairdressing and beauty salon managers and proprietors		
			1234	Shopkeepers and wholesale/retail dealers		
			1235	Recycling and refuse disposal managers		
			1239	Managers and proprietors in other services n.e.c.		
				**PROFESSIONAL OCCUPATIONS**		
	21			**SCIENCE, RESEARCH, ENGINEERING AND TECHNOLOGY PROFESSIONALS**		X
		211		**Natural and Social Science Professionals**	X	
			2111	Chemists		
			2112	Biological scientists and biochemists		
			2113	Physicists, geologists and meteorologists		
		212		**Engineering Professionals**	X	

			2121	Civil engineers		
			2122	Mechanical engineers		
			2123	Electrical engineers		
			2124	Electronics engineers		
			2125	Chemical engineers		
			2126	Design and development engineers		
			2127	Production and process engineers		
			2128	Planning and quality control engineers		
			2129	Engineering professionals n.e.c.		
		**213**		**Information And Communication Technology Professionals**	X	
			2131	IT strategy and planning professionals		
			2132	Software professionals		
	22			**HEALTH PROFESSIONALS**		
		**221**		**Health professionals**	X	X
			2211	Medical practitioners		
			2212	Psychologists		
			2213	Pharmacists/pharmacologists		
			2214	Ophthalmic opticians		
			2215	Dental practitioners		
			2216	Veterinarians		
	**23**			**TEACHING AND RESEARCH PROFESSIONALS**		
		**231**		**Teaching Professionals**	X	X
			2311	Higher education teaching professionals		
			2312	Further education teaching professionals		
			2313	Education officers, school inspectors		
			2314	Secondary education teaching professionals		
			2315	Primary and nursery education teaching professionals		
			2316	Special needs education teaching professionals		
			2317	Registrars and senior administrators of educational establishments		
			2319	Teaching professionals n.e.c.		
		**232**		**Research Professional**	X	X
			2321	Scientific researchers		
			2322	Social science researchers		
			2329	Researchers n.e.c.		
	24			BUSINESS AND PUBLIC SERVICE PROFESSIONALS	X	X
		**241**		**Legal Professionals**		
			2411	Solicitors and lawyers, judges and coroners		
			2419	Legal professionals n.e.c.		
		**242**		**Business And Statistical Professionals**		
			2421	Chartered and certified accountants		
			2422	Management accountants		
			2423	Management consultants, actuaries, economists and statisticians		
		**243**		**Architects, Town Planners, Surveyors**		

			2431	Architects		
			2432	Town planners		
			2433	Quantity surveyors		
			2434	Chartered surveyors (not quantity surveyors)		
		**244**		**Public Service Professionals**		
			2441	Public service administrative professionals		
			2442	Social workers		
			2443	Probation officers		
			2444	Clergy		
		**245**		**Librarians And Related Professionals**		
			2451	Librarians		
			2452	Archivists and curators		
3				**ASSOCIATE PROFESSIONAL AND TECHNICAL OCCUPATIONS**		
	**31**			**SCIENCE AND TECHNOLOGY ASSOCIATE PROFESSIONALS**		X
		**311**		**Science And Engineering Technicians**	X	
			3111	Laboratory technicians		
			3112	Electrical/electronics technicians		
			3113	Engineering technicians		
			3114	Building and civil engineering technicians		
			3115	Quality assurance technicians		
			3119	Science and engineering technicians n.e.c.		
		**312**		**Draughtspersons And Building Inspectors**	X	
			3121	Architectural technologists and town planning technicians		
			3122	Draughtspersons		
			3123	Building inspectors		
		**313**		**IT Service Delivery Occupations**	X	
			3131	IT operations technicians		
			3132	IT user support technicians		
	32			HEALTH AND SOCIAL WELFARE ASSOCIATE PROFESSIONALS		
		**321**		**Health Associate Professionals**	X	X
			3211	Nurses		
			3212	Midwives		
			3213	Paramedics		
			3214	Medical radiographers		
			3215	Chiropodists		
			3216	Dispensing opticians		
			3217	Pharmaceutical dispensers		
			3218	Medical and dental technicians		
		**322**		**Therapists**	X	X
			3221	Physiotherapists		
			3222	Occupational therapists		
			3223	Speech and language therapists		

			3229	Therapists n.e.c.		
		**323**		**Social Welfare Associate Professionals**	X	X
			3231	Youth and community workers		
			3232	Housing and welfare officers		
	33			PROTECTIVE SERVICE OCCUPATIONS		
		**331**		**Protective Service Occupations**	X	X
			3311	NCOs and other ranks		
			3312	Police officers (sergeant and below)		
			3313	Fire service officers (leading fire officer and below)		
			3314	Prison service officers (below principal officer)		
			3319	Protective service associate professionals n.e.c.		
	34			CULTURE, MEDIA AND SPORTS OCCUPATIONS	X	X
		**341**		**Artistic And Literary Occupations**		
			3411	Artists		
			3412	Authors, writers		
			3413	Actors, entertainers		
			3414	Dancers and choreographers		
			3415	Musicians		
			3416	Arts officers, producers and directors		
		**342**		**Design Associate Professionals**		
			3421	Graphic designers		
			3422	Product, clothing and related designers		
		**343**		**Media Associate Professionals**		
			3431	Journalists, newspaper and periodical editors		
			3432	Broadcasting associate professionals		
			3433	Public relations officers		
			3434	Photographers and audio-visual equipment operators		
		**344**		**Sports And Fitness Occupations**		
			3441	Sports players		
			3442	Sports coaches, instructors and officials		
			3443	Fitness instructors		
			3449	Sports and fitness occupations n.e.c.		
	35			BUSINESS AND PUBLIC SERVICE ASSOCIATE PROFESSIONALS		X
		**351**		**Transport Associate Professionals**	X	
			3511	Air traffic controllers		
			3512	Aircraft pilots and flight engineers		
			3513	Ship and hovercraft officers		
			3514	Train drivers		
		**352**		**Legal Associate Professionals**	X	
			3520	Legal associate professionals		
		**353**		**Business And Finance Associate Professionals**	X	
			3531	Estimators, valuers and assessors		
			3532	Brokers		
			3533	Insurance underwriters		

			3534	Finance and investment analysts/advisers		
			3535	Taxation experts		
			3536	Importers, exporters		
			3537	Financial and accounting technicians		
			3539	Business and related associate professionals n.e.c.		
		**354**		**Sales And Related Associate Professionals**	X	
			3541	Buyers and purchasing officers		
			3542	Sales representatives		
			3543	Marketing associate professionals		
			3544	Estate agents, auctioneers		
		**355**		**Conservation Associate Professionals**	X	
			3551	Conservation and environmental protection officers		
			3552	Countryside and park rangers		
		**356**		**Public Service And Other Associate Professionals**	X	
			3561	Public service associate professionals		
			3562	Personnel and industrial relations officers		
			3563	Vocational and industrial trainers and instructors		
			3564	Careers advisers and vocational guidance specialists		
			3565	Inspectors of factories, utilities and trading standards		
			3566	Statutory examiners		
			3567	Occupational hygienists and safety officers (health and safety)		
			3568	Environmental health officers		
				ADMINISTRATIVE AND SECRETARIAL OCCUPATIONS		
4						
	41			ADMINISTRATIVE OCCUPATIONS		
		**411**		**Administrative Occupations: Government And Related Organisations**	X	X
			4111	Civil Service executive officers		
			4112	Civil Service administrative officers and assistants		
			4113	Local government clerical officers and assistants		
			4114	Officers of non-governmental organisations		
		**412**		**Administrative Occupations: Finance**	X	X
			4121	Credit controllers		
			4122	Accounts and wages clerks, book-keepers, other financial clerks		
			4123	Counter clerks		
		**413**		**Administrative Occupations: Records**	X	X
			4131	Filing and other records assistants/clerks		
			4132	Pensions and insurance clerks		
			4133	Stock control clerks		
			4134	Transport and distribution clerks		

			4135	Library assistants/clerks		
			4136	Database assistants/clerks		
			4137	Market research interviewers		
		**414**		**Administrative Occupations: Communications**	X	X
			4141	Telephonists		
			4142	Communication operators		
		**415**		**Administrative Occupations: General**	X	X
			4150	General office assistants/clerks		
	42			SECRETARIAL AND RELATED OCCUPATIONS		
		**421**		**Secretarial And Related Occupations**	X	X
			4211	Medical secretaries		
			4212	Legal secretaries		
			4213	School secretaries		
			4214	Company secretaries		
			4215	Personal assistants and other secretaries		
			4216	Receptionists		
			4217	Typists		
5				SKILLED TRADES OCCUPATIONS		
	51			SKILLED AGRICULTURAL TRADES		
		**511**		**Agricultural Trades**	X	X
			5111	Farmers		
			5112	Horticultural trades		
			5113	Gardeners and groundsmen/groundswomen		
			5119	Agricultural and fishing trades n.e.c.		
	52			SKILLED METAL AND ELECTRICAL TRADES		X
		**521**		**Metal Forming, Welding And Related Trades**	X	
			5211	Smiths and forge workers		
			5212	Moulders, core makers, die casters		
			5213	Sheet metal workers		
			5214	Metal plate workers, shipwrights, riveters		
			5215	Welding trades		
			5216	Pipe fitters		
		**522**		**Metal Machining, Fitting And Instrument Making Trades**	X	
			5221	Metal machining setters and setter-operators		
			5222	Tool makers, tool fitters and markers-out		
			5223	Metal working production and maintenance fitters		
			5224	Precision instrument makers and repairers		
		**523**		**Vehicle Trades**	X	
			5231	Motor mechanics, auto engineers		
			5232	Vehicle body builders and repairers		
			5233	Auto electricians		
			5234	Vehicle spray painters		
		**524**		**Electrical Trades**	X	

			5241	Electricians, electrical fitters		
			5242	Telecommunications engineers		
			5243	Lines repairers and cable jointers		
			5244	TV, video and audio engineers		
			5245	Computer engineers, installation and maintenance		
			5249	Electrical/electronics engineers n.e.c.		
	53			SKILLED CONSTRUCTION AND BUILDING TRADES		X
		**531**		**Construction Trades**	X	
			5311	Steel erectors		
			5312	Bricklayers, masons		
			5313	Roofers, roof tilers and slaters		
			5314	Plumbers, heating and ventilating engineers		
			5315	Carpenters and joiners		
			5316	Glaziers, window fabricators and fitters		
			5319	Construction trades n.e.c.		
		**532**		**Building Trades**	X	
			5321	Plasterers		
			5322	Floorers and wall tilers		
			5323	Painters and decorators		
	54			TEXTILES, PRINTING AND OTHER SKILLED TRADES		
		**541**		**Textiles And Garments Trades**	X	X
			5411	Weavers and knitters		
			5412	Upholsterers		
			5413	Leather and related trades		
			5414	Tailors and dressmakers		
			5419	Textiles, garments and related trades n.e.c.		
		**542**		**Printing Trades**	X	X
			5421	Originators, compositors and print preparers		
			5422	Printers		
			5423	Bookbinders and print finishers		
			5424	Screen printers		
		**543**		**Food Preparation Trades**	X	X
			5431	Butchers, meat cutters		
			5432	Bakers, flour confectioners		
			5433	Fishmongers, poultry dressers		
			5434	Chefs, cooks		
		**549**		**Skilled Trades n. e. c.**	X	X
			5491	Glass and ceramics makers, decorators and finishers		
			5492	Furniture makers, other craft woodworkers		
			5493	Pattern makers (moulds)		
			5494	Musical instrument makers and tuners		
			5495	Goldsmiths, silversmiths, precious stone workers		
			5496	Floral arrangers, florists		
			5499	Hand craft occupations n.e.c.		

6				PERSONAL SERVICE OCCUPATIONS		
	61			CARING PERSONAL SERVICE OCCUPATIONS		
		**611**		**Healthcare And Related Personal Services**	X	X
			6111	Nursing auxiliaries and assistants		
			6112	Ambulance staff (excluding paramedics)		
			6113	Dental nurses		
			6114	Houseparents and residential wardens		
			6115	Care assistants and home carers		
		**612**		**Childcare And Related Personal Services**	X	X
			6121	Nursery nurses		
			6122	Childminders and related occupations		
			6123	Playgroup leaders/assistants		
			6124	Educational assistants		
		**613**		**Animal Care Services**	X	X
			6131	Veterinary nurses and assistants		
			6139	Animal care occupations n.e.c.		
	62			LEISURE AND OTHER PERSONAL SERVICE OCCUPATIONS		
		**621**		**Leisure And Travel Service Occupations**	X	X
			6211	Sports and leisure assistants		
			6212	Travel agents		
			6213	Travel and tour guides		
			6214	Air travel assistants		
			6215	Rail travel assistants		
			6219	Leisure and travel service occupations n.e.c.		
		**622**		**Hairdressers And Related Occupations**	X	X
			6221	Hairdressers, barbers		
			6222	Beauticians and related occupations		
		**623**		**Housekeeping Occupations**	X	X
			6231	Housekeepers and related occupations		
			6232	Caretakers		
		**629**		**Personal Services Occupations n. e. c.**	X	X
			6291	Undertakers and mortuary assistants		
			6292	Pest control officers		
7				SALES AND CUSTOMER SERVICE OCCUPATIONS		
	71			SALES OCCUPATIONS		
		**711**		**Sales Assistants And Retail Cashiers**	X	X
			7111	Sales and retail assistants		
			7112	Retail cashiers and check-out operators		
			7113	Telephone salespersons		
		**712**		**Sales Related Occupations**	X	X
			7121	Collector salespersons and credit agents		
			7122	Debt, rent and other cash collectors		

			7123	Roundsmen/women and van salespersons		
			7124	Market and street traders and assistants		
			7125	Merchandisers and window dressers		
			7129	Sales related occupations n.e.c.		
	72			CUSTOMER SERVICE OCCUPATIONS		
		**721**		**Customer Service Occupations**	X	X
			7211	Call centre agents/operators		
			7212	Customer care occupations		
8				PROCESS, PLANT AND MACHINE OPERATIVES		
	81			PROCESS, PLANT AND MACHINE OPERATIVES		
		**811**		**Process Operatives**	X	X
			8111	Food, drink and tobacco process operatives		
			8112	Glass and ceramics process operatives		
			8113	Textile process operatives		
			8114	Chemical and related process operatives		
			8115	Rubber process operatives		
			8116	Plastics process operatives		
			8117	Metal making and treating process operatives		
			8118	Electroplaters		
			8119	Process operatives n.e.c.		
		**812**		**Plant And Machine Operatives**	X	X
			8121	Paper and wood machine operatives		
			8122	Coal mine operatives		
			8123	Quarry workers and related operatives		
			8124	Energy plant operatives		
			8125	Metal working machine operatives		
			8126	Water and sewerage plant operatives		
			8129	Plant and machine operatives n.e.c.		
		**813**		**Assemblers And Routine Operatives**	X	X
			8131	Assemblers (electrical products)		
			8132	Assemblers (vehicles and metal goods)		
			8133	Routine inspectors and testers		
			8134	Weighers, graders, sorters		
			8135	Tyre, exhaust and windscreen fitters		
			8136	Clothing cutters		
			8137	Sewing machinists		
			8138	Routine laboratory testers		
			8139	Assemblers and routine operatives n.e.c.		
		**814**		**Construction Operatives**	X	X
			8141	Scaffolders, stagers, riggers		
			8142	Road construction operatives		
			8143	Rail construction and maintenance operatives		
			8149	Construction operatives n.e.c.		
	82			TRANSPORT AND MOBILE MACHINE DRIVERS AND OPERATIVES		

		821		**Transport Drivers And Operatives**	X	X
			8211	Heavy goods vehicle drivers		
			8212	Van drivers		
			8213	Bus and coach drivers		
			8214	Taxi, cab drivers and chauffeurs		
			8215	Driving instructors		
			8216	Rail transport operatives		
			8217	Seafarers (merchant navy); barge, lighter and boat operatives		
			8218	Air transport operatives		
			8219	Transport operatives n.e.c.		
		822		**Mobile Machine Drivers And Operatives**	X	X
			8221	Crane drivers		
			8222	Fork-lift truck drivers		
			8223	Agricultural machinery drivers		
			8229	Mobile machine drivers and operatives n.e.c.		
9				ELEMENTARY OCCUPATIONS		
	91			ELEMENTARY TRADES, PLANT AND STORAGE RELATED OCCUPATIONS		
		911		**Elementary Agricultural Occupations**	X	X
			9111	Farm workers		
			9112	Forestry workers		
			9119	Fishing and agriculture related occupations n.e.c.		
		912		**Elementary Construction Occupations**	X	X
			9121	Labourers in building and woodworking trades		
			9129	Labourers in other construction trades n.e.c.		
		913		**Elementary Process Plant Occupations**	X	X
			9131	Labourers in foundries		
			9132	Industrial cleaning process occupations		
			9133	Printing machine minders and assistants		
			9134	Packers, bottlers, canners, fillers		
			9139	Labourers in process and plant operations n.e.c.		
		914		**Elementary Goods Storage Occupations**	X	X
			9141	Stevedores, dockers and slingers		
			9149	Other goods handling and storage occupations n.e.c.		
	92			ELEMENTARY ADMINISTRATION AND SERVICE OCCUPATIONS		
		921		**Elementary Administration Occupations**	X	X
			9211	Postal workers, mail sorters, messengers, couriers		
			9219	Elementary office occupations n.e.c.		
		922		**Elementary Personal Services Occupations**	X	X
			9221	Hospital porters		
			9222	Hotel porters		
			9223	Kitchen and catering assistants		

			9224	Waiters, waitresses		
			9225	Bar staff		
			9226	Leisure and theme park attendants		
			9229	Elementary personal services occupations n.e.c.		
		**923**		**Elementary Cleaning Occupations**	X	X
			9231	Window cleaners		
			9232	Road sweepers		
			9233	Cleaners, domestics		
			9234	Launderers, dry cleaners, pressers		
			9235	Refuse and salvage occupations		
			9239	Elementary cleaning occupations n.e.c.		
		**924**		**Elementary Security Occupations**	X	X
			9241	Security guards and related occupations		
			9242	Traffic wardens		
			9243	School crossing patrol attendants		
			9244	School mid-day assistants		
			9245	Car park attendants		
			9249	Elementary security occupations n.e.c.		
		**925**		**Elementary Sales Occupations**	X	X
			9251	Shelf fillers		
			9259	Elementary sales occupations n.e.c.		

Appendix Table 2a: Mortality rates by occupation in Scotland in men

Employment group	SOC code*	Person-Years	EASR	Low 95% CI	High 95% CI	Rank
Corporate managers and directors	11	5312	244	117	372	9
Production Managers	112	20347	243	182	305	8
Functional Managers	113	21323	261	172	351	11
Financial Institution And Office Managers	115	6876	321	191	451	15
Managers In Distribution, Storage And Retailing	116	13312	363	252	474	23
Protective Service Officers	117	2858	436	165	707	30
Managers and proprietors in agriculture and services	12	10763	353	242	464	22
Managers And Proprietors In Other Service Industries	123	15127	333	241	426	18
Engineering Professionals	212	14990	333	234	432	17
Information And Communication Technology Professionals	213	10171	214	81	347	2
Health professionals	221	6685	145	50	240	1
Teaching Professionals	231	16617	219	158	281	4
Research Professional	232	2740	328	26	630	16
Business and public service professionals	24	18640	218	149	287	3
Science And Engineering Technicians	311	13016	314	206	422	14
Draughtspersons And Building Inspectors	312	3318	494	212	777	38
IT Service Delivery Occupations	313	4575	224	0	456	5
Health Associate Professionals	321	4593	438	220	657	31
Social Welfare Associate Professionals	323	3278	372	141	603	24
Protective Service Occupations	331	17173	386	255	517	26
Culture, media and sports occupations	34	10630	307	187	427	13
Transport Associate Professionals	351	3690	339	136	543	19
Business And Finance Associate Professionals	353	9666	229	125	333	6
Sales And Related Associate Professionals	354	10799	235	140	329	7
Public Service And Other Associate Professionals	356	7539	379	242	517	25
Administrative Occupations: Government And Related Organisations	411	6005	521	343	699	45
Administrative Occupations: Finance	412	8347	303	161	446	12
Administrative Occupations: Records	413	10452	346	220	472	20
Administrative Occupations: Communications	414	1241	724	39	1410	56
Administrative Occupations: General	415	6396	430	242	618	27
Secretarial And Related Occupations	421					
Agricultural Trades	511	18949	447	350	544	33
Metal Forming, Welding And Related Trades	521	9910	507	370	643	41
Metal Machining, Fitting And Instrument Making Trades	522	21221	431	344	518	28
Vehicle Trades	523	10771	351	231	471	21
Electrical Trades	524	22986	462	371	552	35
Construction Trades	531	35967	501	425	577	39
Building Trades	532	11551	513	380	646	43
Textiles And Garments Trades	541					
Printing Trades	542	2804	638	315	962	53
Food Preparation Trades	543	12822	485	351	619	37
Skilled Trades n. e. c.	549	3415	461	219	703	34
Healthcare And Related Personal Services	611	6333	434	266	603	29
Animal Care Services	613	479	1125	127	2123	61
Leisure And Travel Service Occupations	621	3368	721	377	1065	54
Hairdressers And Related Occupations	622					
Housekeeping Occupations	623	3591	568	318	818	48
Sales Assistants And Retail Cashiers	711	14228	444	283	605	32
Sales Related Occupations	712	4458	509	303	715	42
Customer Service Occupations	721	5608	247	49	444	10
Process Operatives	811	13674	476	359	592	36
Plant And Machine Operatives	812	16897	521	410	631	44
Assemblers And Routine Operatives	813	13354	630	475	785	52
Construction Operatives	814	10330	623	472	774	51

Transport Drivers And Operatives	821	42023	503	440	566	40
Mobile Machine Drivers And Operatives	822	9897	559	410	708	47
Elementary Agricultural Occupations	911	7130	572	390	754	49
Elementary Construction Occupations	912	13061	937	771	1103	60
Elementary Process Plant Occupations	913	13085	829	666	992	59
Elementary Goods Storage Occupations	914	16057	619	486	752	50
Elementary Administration Occupations	921	9548	545	390	700	46
Elementary Personal Services Occupations	922	11572	724	520	927	55
Elementary Cleaning Occupations	923	10567	770	605	934	58
Elementary Security Occupations	924	7543	740	560	919	57
Elementary Sales Occupations	925					
No occupation reported		52255	1242	1131	1352	
All those reporting an occupation§		680710	437	421	454	

EASR = European age-standardised mortality rates per 100,000 person-years

*Standard Occupational Classification (SOC) codes have been modified for disclosure control purposes. See Appendix Table 1 for details.

§Note that this includes not only SOC categories listed, but also small occupational groups that have been suppressed for disclosure control (equating to a total of 1.5% of all person-years). Therefore, person-years for the SOC codes listed do not total to the last two rows.

Source: Scottish Longitudinal Study

Appendix Table 2b: Mortality rates by occupation in Scotland in women

Employment group	SOC code*	Person-Years	EASR	Low 95% CI	High 95% CI	Rank
Corporate managers and directors	11	39799	178	131	225	5
Managers and proprietors in agriculture and services	12	11323	236	147	325	18
Managers And Proprietors In Other Service Industries	123	9257	243	148	338	21
Science, research, engineering and technology professionals	21	6091	429	24	833	37
Health professionals	221	6144	215	63	366	11
Teaching Professionals	231	38283	160	122	197	4
Research Professional	232					
Business and public service professionals	24	12492	201	109	292	8
Science and technology associate professionals	31	7445	220	100	341	12
Health Associate Professionals	321	34289	241	186	296	20
Therapists	322	4924	148	28	269	2
Social Welfare Associate Professionals	323	10220	202	110	295	9
Protective Service Occupations	331	2481	570	42	1097	42
Culture, media and sports occupations	34	8413	203	83	323	10
Business and public service associate professionals	35	28254	200	131	269	7
Administrative Occupations: Government And Related Organisations	411	13429	238	154	321	19
Administrative Occupations: Finance	412	30513	249	190	308	22
Administrative Occupations: Records	413	17717	222	147	297	13
Administrative Occupations: Communications	414	4428	273	118	428	25
Administrative Occupations: General	415	39050	226	177	276	15
Secretarial And Related Occupations	421	42318	223	180	266	14
Agricultural Trades	511	2138	118	15	221	1
Skilled metal and electrical trades	52	1786	387	80	694	34
Skilled construction and building trades	53					
Textiles And Garments Trades	541	1931	478	200	756	39
Printing Trades	542	1446	330	38	622	28
Food Preparation Trades	543	11025	422	299	546	35
Skilled Trades n. e. c.	549					
Healthcare And Related Personal Services	611	46928	284	237	330	26
Childcare And Related Personal Services	612	22117	232	156	309	17
Leisure And Travel Service Occupations	621	6846	228	99	357	16
Hairdressers And Related Occupations	622	9776	356	185	526	33
Housekeeping Occupations	623	5033	254	129	380	23
Sales Assistants And Retail Cashiers	711	71571	308	264	351	27
Sales Related Occupations	712	4023	186	46	325	6
Customer Service Occupations	721	14348	158	83	233	3
Process Operatives	811	9176	338	218	457	29
Plant And Machine Operatives	812	2135	663	320	1006	43
Assemblers And Routine Operatives	813	21991	494	399	588	40
Transport Drivers And Operatives	821	2587	257	52	462	24
Elementary Agricultural Occupations	911					
Elementary Process Plant Occupations	913	11075	467	338	595	38
Elementary Goods Storage Occupations	914	2256	528	189	867	41
Elementary Administration Occupations	921	5060	354	180	528	32
Elementary Personal Services Occupations	922	35905	346	276	415	31
Elementary Cleaning Occupations	923	44486	426	371	481	36
Elementary Security Occupations	924	3801	339	178	500	30
Elementary Sales Occupations	925					
No occupation reported		64710	695	628	762	
All those reporting an occupation§		716950	277	264	290	

EASR = European age-standardised mortality rates per 100,000 person-years

*Standard Occupational Classification (SOC) codes have been modified for disclosure control purposes. See Appendix Table 1 for details.

§Note that this includes not only SOC categories listed, but also small occupational groups that have been suppressed for disclosure control (equating to a total of 1.6% of all person-years). Therefore, person-years for the SOC codes listed do not total to the last two rows.

Source: Scottish Longitudinal Study

Appendix Table 3a: Mortality rates by occupation in Northern Ireland in men

Employment group	SOC code*	Person-Years	EASR	Low 95% CI	High 95% CI	Rank
Corporate managers and directors	11	380331	233	217	249	6
Managers and proprietors in agriculture and services	12	55930	349	298	400	25
Managers And Proprietors In Other Service Industries	123	89047	258	227	288	9
Natural and Social Science Professionals	211	11302	318	216	420	14
Engineering Professionals	212	64710	200	159	240	3
Information And Communication Technology Professionals	213	47305	243	140	346	7
Health professionals	221	40230	137	98	176	1
Teaching Professionals	231	126477	203	181	225	4
Research Professional	232	14439	258	156	359	8
Business and public service professionals	24	107453	182	155	208	2
Associate professional and technical occupations	3	514830	285	268	302	10
Administrative Occupations: Government And Related Organisations	411	110582	338	305	372	19
Administrative Occupations: Finance	412	58421	339	285	393	20
Administrative Occupations: Records	413	44727	315	254	376	13
Administrative Occupations: Communications	414	5381	398	214	583	35
Administrative Occupations: General	415	93181	346	299	392	24
Secretarial And Related Occupations	421	8339	222	115	328	5
Agricultural Trades	511	174085	287	263	311	11
Metal Forming, Welding And Related Trades	521	62668	418	361	476	41
Metal Machining, Fitting And Instrument Making Trades	522	128242	349	315	383	26
Vehicle Trades	523	70657	334	284	384	17
Electrical Trades	524	140711	318	286	351	15
Construction Trades	531	300939	341	317	365	22
Building Trades	532	103712	407	365	449	38
Textiles And Garments Trades	541	10779	337	225	449	18
Printing Trades	542	11865	351	228	473	27
Food Preparation Trades	543	81829	418	360	476	40
Skilled Trades n. e. c.	549	30695	380	295	465	34
Healthcare And Related Personal Services	611	32005	407	328	486	37
Childcare And Related Personal Services	612	3058	471	159	784	47
Animal Care Services	613	2704	614	292	936	51
Leisure And Travel Service Occupations	621	11486	427	275	580	42
Hairdressers And Related Occupations	622	6260	322	136	507	16
Housekeeping Occupations	623	22329	359	292	426	30
Sales Assistants And Retail Cashiers	711	101906	351	297	404	28
Sales Related Occupations	712	27734	302	239	366	12
Customer Service Occupations	721	14179	412	235	589	39
Process Operatives	811	136199	405	367	443	36
Plant And Machine Operatives	812	109011	339	301	378	21
Assemblers And Routine Operatives	813	71774	370	314	426	31
Construction Operatives	814	63498	353	307	400	29
Transport Drivers And Operatives	821	282078	373	351	395	32
Mobile Machine Drivers And Operatives	822	64764	438	386	489	43
Elementary Agricultural Occupations	911	41969	449	379	519	45
Elementary Construction Occupations	912	122572	522	481	562	49
Elementary Process Plant Occupations	913	90871	539	486	593	50
Elementary Goods Storage Occupations	914	100886	439	393	484	44

Elementary Administration Occupations	921	52375	344	286	402	23
Elementary Personal Services Occupations	922	66949	639	563	714	52
Elementary Cleaning Occupations	923	55962	468	410	526	46
Elementary Security Occupations	924	54217	505	449	560	48
Elementary Sales Occupations	925	9258	374	170	577	33

EASR = European age-standardised mortality rates per 100,000 person-years

*Standard Occupational Classification (SOC) codes have been modified for disclosure control purposes. See Appendix Table 1 for details.

Source: Northern Ireland Mortality Study

Appendix Table 3b: Mortality rates by occupation in Northern Ireland in women

Employment group	SOC code*	Person-Years	EASR	Low 95% CI	High 95% CI	Rank
Corporate managers and directors	11	212609	166	146	187	7
Managers and proprietors in agriculture and services	12	50035	230	184	276	23
Managers And Proprietors In Other Service Industries	123	50206	211	172	249	16
Science, research, engineering and technology professionals	21	29248	155	82	227	5
Health professionals	221	33077	95	45	144	1
Teaching Professionals	231	271711	134	120	147	2
Research Professional	232	13904	205	85	326	15
Business and public service professionals	24	68042	145	108	181	3
Associate professionals and technical occupations	3	533249	177	164	190	9
Administrative Occupations: Government And Related Organisations	411	117934	198	170	225	14
Administrative Occupations: Finance	412	193982	196	173	219	13
Administrative Occupations: Records	413	87098	173	141	205	8
Administrative Occupations: Communications	414	22074	286	215	356	34
Administrative Occupations: General	415	270682	223	202	243	18
Secretarial And Related Occupations	421	274263	186	169	204	11
Agricultural Trades	511	14029	239	161	318	26
Skilled metal and electrical trades	52	14197	303	210	396	37
Skilled construction and building trades	53	6231	279	149	408	33
Textiles And Garments Trades	541	13885	331	249	413	40
Printing Trades	542	6202	181	74	288	10
Food Preparation Trades	543	93290	250	216	283	27
Skilled Trades n. e. c.	549	7858	337	194	479	41
Healthcare And Related Personal Services	611	269033	227	209	246	21
Childcare And Related Personal Services	612	145457	158	134	183	6
Leisure And Travel Service Occupations	621	26611	220	146	294	17
Hairdressers And Related Occupations	622	77852	230	180	279	24
Housekeeping Occupations	623	28147	256	203	309	29
Sales Assistants And Retail Cashiers	711	473183	234	218	250	25
Sales Related Occupations	712	15244	154	86	222	4
Customer Service Occupations	721	36599	227	133	320	19
Process Operatives	811	124130	319	288	350	38
Plant And Machine Operatives	812	32976	293	227	359	36
Assemblers And Routine Operatives	813	192729	324	298	350	39
Transport Drivers And Operatives	821	11733	291	177	404	35
Elementary Agricultural Occupations	911	8532	193	97	289	12
Elementary Process Plant Occupations	913	79215	339	299	380	42
Elementary Goods Storage Occupations	914	12120	227	130	324	20
Elementary Administration Occupations	921	28668	276	209	344	31
Elementary Personal Services Occupations	922	194105	268	243	292	30
Elementary Cleaning Occupations	923	253449	277	258	295	32
Elementary Security Occupations	924	36509	254	206	301	28
Elementary Sales Occupations	925	17610	228	139	317	22

EASR = European age-standardised mortality rates per 100,000 person-years

*Standard Occupational Classification (SOC) codes have been modified for disclosure control purposes. See Appendix Table 1 for details.

Source: Northern Ireland Mortality Study

Appendix Table 4: Comparison of mortality rates by occupation between men and women in England/Wales for selected occupational groups

		Males					Females				
Employment group	SOC code*	Person-Years	EASR	Low 95% CI	High 95% CI	Rank	Person-Years	EASR	Low 95% CI	High 95% CI	Rank
MANAGERS AND PROPRIETORS IN AGRICULTURE AND SERVICES*	12	23348	450	364	536	24	23892	397	318	476	34
Managers And Proprietors In Other Service Industries	123	36544	333	278	387	5	23180	227	162	291	11
Health professionals	221	12873	225	145	304	1	10099	213	107	318	5
Teaching Professionals	231	36736	262	210	313	3	79871	180	152	208	3
BUSINESS AND PUBLIC SERVICE PROFESSIONALS	24	41276	228	182	274	2	25728	159	104	214	2
Health Associate Professionals	321	9248	288	171	404	4	58260	230	191	270	13
Social Welfare Associate Professionals	323	5084	334	166	501	6	13795	236	153	318	15
CULTURE, MEDIA AND SPORTS OCCUPATIONS	34	32202	350	273	428	10	26903	133	78	188	1
Administrative Occupations: Government And Related Organisations	411	11250	373	268	479	12	21080	263	192	334	18
Administrative Occupations: Finance	412	23118	347	265	429	8	72532	264	223	305	19
Administrative Occupations: Records	413	20129	418	318	517	19	37807	333	271	394	27
Administrative Occupations: Communications	414	2204	604	246	962	35	6727	368	233	502	30
Administrative Occupations: General	415	16102	461	342	581	25	76601	233	198	268	14
Secretarial And Related Occupations	421	4784	348	187	510	9	112533	218	192	244	7
Agricultural Trades	511	23913	402	325	479	16	4776	251	114	389	16
Textiles And Garments Trades	541	3809	569	330	808	33	3810	483	266	700	36
Printing Trades	542	7140	403	244	562	17	3557	256	104	409	17
Food Preparation Trades	543	23061	439	335	542	22	18319	217	153	281	6
Skilled Trades n. e. c.	549	9553	391	259	524	15	4170	380	175	585	31
Healthcare And Related Personal Services	611	11400	413	287	539	18	88120	290	255	325	22
Leisure And Travel Service Occupations	621	6145	345	165	524	7	12344	222	116	328	10
Hairdressers And Related Occupations	622	2755	492	226	759	26	21910	209	142	277	4
Housekeeping Occupations	623	5202	567	402	732	32	8417	306	204	407	25
Sales Assistants And Retail Cashiers	711	33239	429	331	526	21	137985	305	275	336	24

Sales Related Occupations	712	9552	368	251	485	11	9852	265	156	375	20
Customer Service Occupations	721	8397	385	198	573	13	25489	230	153	307	12
Process Operatives	811	30195	529	445	614	28	17107	346	259	433	28
Plant And Machine Operatives	812	38091	423	360	487	20	8707	517	370	663	37
Assemblers And Routine Operatives	813	25176	501	410	593	27	34561	386	324	448	33
Transport Drivers And Operatives	821	82677	445	401	489	23	6560	279	132	426	21
Elementary Process Plant Occupations	913	32513	672	576	767	37	30212	405	334	476	35
Elementary Goods Storage Occupations	914	37887	539	459	619	29	7984	220	104	336	9
Elementary Administration Occupations	921	22354	389	305	473	14	12940	383	275	492	32
Elementary Personal Services Occupations	922	21359	650	510	790	36	59492	314	265	363	26
Elementary Cleaning Occupations	923	20096	592	487	696	34	65159	352	311	394	29
Elementary Security Occupations	924	15272	544	433	655	30	16424	301	222	380	23
Elementary Sales Occupations	925	3571	556	209	903	31	6775	218	93	344	8
No occupation reported		39614	1189	1014	1364		83370	587	523	651	
All those reporting an occupation*		1457772	384	374	394		1485036	263	255	272	

EASR = European age-standardised mortality rates per 100,000 person-years

*Standard Occupational Classification (SOC) codes have been modified for disclosure control purposes. See Appendix Table 1 for details.

Source: ONS Longitudinal Study

Appendix Table 5a: Expected change in deaths in England/Wales among working age men if mortality rates by occupation for Scotland applied §

Employment group	SOC code*	Difference between England and Scotland	95% CI	p value	Expected change in deaths in England	95% CI
Corporate managers and directors	11	6	-142, 155	0.936	-8.7	-220, 203
Production Managers	112	21	-57, 97	0.595	-119	-552, 325
Functional Managers	113	-28	-125, 71	0.567	176	-443, 781
Financial Institution And Office Managers	115	-86	-235, 58	0.245	188	-126, 512
Managers In Distribution, Storage And Retailing	116	-87	-207, 34	0.154	397	-154, 944
Protective Service Officers	117	-111	-410, 183	0.478	72.2	-119, 267
Managers and proprietors in agriculture and services	12	97	-42, 238	0.169	-231	-566, 101
Managers And Proprietors In Other Service Industries	123	-1.2	-110, 105	0.986	4.5	-393, 410
Engineering Professionals	212	-51	-165, 62	0.38	187	-226, 601
Information And Communication Technology Professionals	213	52	-104, 209	0.51	-169	-671, 334
Health professionals	221	79	-45, 202	0.21	-104	-266, 60
Teaching Professionals	231	43	-36, 124	0.293	-161	-464, 134
Business and public service professionals	24	10	-72, 92	0.815	-43	-387, 302
Science And Engineering Technicians	311	-43	-171, 88	0.513	103	-208, 405
Draughtspersons And Building Inspectors	312	-213	-536, 105	0.19	109	-54, 274
IT Service Delivery Occupations	313	127	-165, 430	0.41	-138	-464, 178
Health Associate Professionals	321	-148	-400, 99	0.236	140	-93, 378
Social Welfare Associate Professionals	323	-37	-327, 251	0.798	19	-130, 170
Protective Service Occupations	331	-123	-276, 30	0.114	361	-87, 813
Culture, media and sports occupations	34	43	-101, 186	0.555	-141	-612, 333
Transport Associate Professionals	351	-49	-324, 229	0.708	25	-119, 168
Business And Finance Associate Professionals	353	39	-86, 164	0.537	-104	-435, 228
Sales And Related Associate Professionals	354	126	4.7, 250	0.043	-347	-687, -13
Public Service And Other Associate Professionals	356	-70	-236, 95	0.413	97	-133, 330
Administrative Occupations: Government And Related Organisations	411	-148	-358, 61	0.156	171	-70, 412
Administrative Occupations: Finance	412	44	-120, 210	0.609	-103	-496, 284

Administrative Occupations: Records	413	71	-90, 231	0.39	-145	-476, 185
Administrative Occupations: Communications	414	-123	-888, 655	0.756	28	-147, 200
Administrative Occupations: General	415	30	-189, 254	0.796	-49	-418, 311
Agricultural Trades	511	-45	-167, 78	0.479	110	-190, 408
Metal Forming, Welding And Related Trades	521	55	-124, 233	0.555	-92	-392, 208
Metal Machining, Fitting And Instrument Making Trades	522	-31	-135, 75	0.56	136	-328, 591
Vehicle Trades	523	106	-47, 258	0.18	-249	-608, 111
Electrical Trades	524	-128	-235, -21	0.019	547	91, 1005
Construction Trades	531	-83	-171, 8.1	0.073	603	-59, 1252
Building Trades	532	-67	-224, 93	0.407	160	-221, 533
Printing Trades	542	-238	-605, 126	0.188	173	-92, 441
Food Preparation Trades	543	-48	-213, 121	0.587	113	-284, 501
Skilled Trades n. e. c.	549	-73	-349, 201	0.611	71	-196, 341
Healthcare And Related Personal Services	611	-20	-233, 190	0.858	23	-221, 271
Leisure And Travel Service Occupations	621	-377	-761, 11	0.059	237	-6.9, 478
Housekeeping Occupations	623	-1	-302, 299	0.996	0.5	-159, 160
Sales Assistants And Retail Cashiers	711	-16	-205, 174	0.869	53	-589, 696
Sales Related Occupations	712	-140	-379, 97	0.245	137	-94, 369
Customer Service Occupations	721	138	-138, 409	0.327	-118	-351, 118
Process Operatives	811	53	-88, 196	0.478	-163	-605, 270
Plant And Machine Operatives	812	-98	-224, 31	0.139	380	-119, 873
Assemblers And Routine Operatives	813	-128	-310, 55	0.161	329	-142, 797
Construction Operatives	814	-117	-302, 66	0.207	195	-110, 500
Transport Drivers And Operatives	821	-58	-136, 18	0.132	492	-148, 1146
Mobile Machine Drivers And Operatives	822	-3.1	-197, 184	0.982	4.6	-276, 295
Elementary Agricultural Occupations	911	50	-208, 309	0.708	-42	-255, 172
Elementary Construction Occupations	912	-235	-433, -37	0.022	552	86, 1016
Elementary Process Plant Occupations	913	-157	-345, 33	0.107	520	-109, 1145
Elementary Goods Storage Occupations	914	-80	-235, 70	0.308	311	-271, 909
Elementary Administration Occupations	921	-154	-330, 21	0.088	352	-49, 753
Elementary Personal Services Occupations	922	-73	-323, 175	0.565	159	-381, 704

Elementary Cleaning Occupations	923	-179	-376, 17	0.072	368	-35, 771
Elementary Security Occupations	924	-196	-407, 13	0.07	306	-20, 634
No occupation reported		-51	-262, 154	0.634	206	-623, 1060
TOTAL					6086	3008, 9175

*Standard Occupational Classification (SOC) codes have been modified for disclosure control purposes. See Appendix Table 1 for details.

§ To assess differences between Scotland and England, Monte Carlo simulation was used to estimate the difference between the rate in Scotland and England. 95% CI and p values were calculated using this method as well. Simulated 95% CI are indicated using brackets.

Source: ONS Longitudinal Study and Scottish Longitudinal Study

Appendix Table 5b: Expected change in deaths in England among working age women if mortality rates by occupation for Scotland applied §

Employment group	SOC code*	Difference between England and Scotland	95% CI	p value	Expected change in deaths in England	95% CI
Corporate managers and directors	11	30	-26, 87	0.29	-319	-911, 269
Managers and proprietors in agriculture and services	12	160	41, 276	0.01	-378	-653, -98
Managers And Proprietors In Other Service Industries	123	-16	-132, 98	0.77	38	-225, 303
Science, research, engineering and technology professionals	21	-252	-670, 159	0.23	335	-211, 890
Health professionals	221	-1	-191, 185	0.99	1	-185, 191
Teaching Professionals	231	20	-27, 67	0.39	-161	-530, 212
Business and public service professionals	24	-42	-150, 64	0.45	108	-162, 382
Science and technology associate professionals	31	-17	-155, 124	0.80	27	-193, 242
Health Associate Professionals	321	-11	-80, 57	0.76	60	-331, 459
Social Welfare Associate Professionals	323	34	-92, 160	0.60	-46	-218, 125
Culture, media and sports occupations	34	-69	-200, 62	0.31	183	-165, 532
Business and public service associate professionals	35	-11	-93, 67	0.79	73	-427, 592
Administrative Occupations: Government And Related Organisations	411	25	-85, 132	0.66	-52	-275, 177
Administrative Occupations: Finance	412	15	-56, 87	0.69	-106	-625, 398
Administrative Occupations: Records	413	111	13, 207	0.03	-416	-775, -47
Administrative Occupations: Communications	414	92	-115, 294	0.38	-61	-195, 76
Administrative Occupations: General	415	6	-56, 69	0.86	-46	-519, 426
Secretarial And Related Occupations	421	-6	-57, 45	0.82	64	-504, 632
Agricultural Trades	511	134	-39, 302	0.13	-63	-143, 18
Skilled metal and electrical trades	52	-19	-379 ,336	0.91	9	-158, 178
Textiles And Garments Trades	541	8	-340, 361	0.97	-3	-136, 128
Printing Trades	542	-73	-397, 251	0.67	26	-88, 140
Food preparation trades	543	-205	-344, -67	0.004	371	122, 623
Healthcare And Related Personal Services	611	7	-51, 66	0.82	-58	-574, 448
Childcare And Related Personal Services	612	-29	-120, 60	0.54	158	-333, 660
Leisure And Travel Service Occupations	621	-6	-173, 167	0.95	7	-204, 211
Hairdressers And Related Occupations	622	-146	-330, 37	0.12	317	-81, 716

Housekeeping Occupations	623	50	-112, 211	0.54	-42	-176, 93
Sales Assistants And Retail Cashiers	711	-2	-55, 51	0.93	32	-690, 752
Sales Related Occupations	712	80	-99, 256	0.37	-78	-250, 96
Customer Service Occupations	721	71	-37, 180	0.19	-179	-454, 94
Process Operatives	811	9	-134, 155	0.91	-15	-262, 226
Plant And Machine Operatives	812	-145	-517, 223	0.44	125	-192, 446
Assemblers And Routine Operatives	813	-108	-221, 1.7	0.06	370	-5.9, 756
Transport Drivers And Operatives	821	22	-232, 271	0.86	-14	-176, 150
Elementary Process Plant Occupations	913	-62	-206, 85	0.41	184	-253, 615
Elementary Goods Storage Occupations	914	-311	-668, 54	0.09	245	-42, 528
Elementary Administration Occupations	921	29	-180, 236	0.78	-38	-302, 230
Elementary Personal Services Occupations	922	-31	-115, 52	0.48	182	-308, 677
Elementary Cleaning Occupations	923	-74	-144, -3.3	0.04	476	21, 924
Elementary Security Occupations	924	-39	-219, 137	0.68	63	-223, 355
No occupation reported		-108	-200, -16	0.02	893	135, 1646
TOTAL					2273	-165, 4688

*Standard Occupational Classification (SOC) codes have been modified for disclosure control purposes. See Appendix Table 1 for details.

§ To assess differences between Scotland and England, Monte Carlo simulation was used to estimate the difference between the rate in Scotland and England. 95% CI and p values were calculated using this method as well. Simulated 95% CI are indicated using brackets.

Source: ONS Longitudinal Study and Scottish Longitudinal Study
